# The STROMICS genome study: deep whole-genome sequencing and analysis of 10K Chinese patients with ischemic stroke reveal complex genetic and phenotypic interplay

**DOI:** 10.1038/s41421-023-00582-8

**Published:** 2023-07-21

**Authors:** Si Cheng, Zhe Xu, Shengzhe Bian, Xi Chen, Yanfeng Shi, Yanran Li, Yunyun Duan, Yang Liu, Jinxi Lin, Yong Jiang, Jing Jing, Zixiao Li, Yilong Wang, Xia Meng, Yaou Liu, Mingyan Fang, Xin Jin, Xun Xu, Jian Wang, Chaolong Wang, Hao Li, Siyang Liu, Yongjun Wang

**Affiliations:** 1 Department of Neurology, Beijing Tiantan Hospital, Capital Medical University, Beijing, China; 2 China National Clinical Research Center for Neurological Diseases, Beijing, China; 3 Changping Laboratory, Beijing, China; 4 Clinical Center for Precision Medicine in Stroke, Capital Medical University, Beijing, China; 5 Center of excellence for Omics Research (CORe), Beijing Tiantan Hospital, Capital Medical University, Beijing, China; 6 School of Public Health (Shenzhen), Sun Yat-sen University, Shenzhen, Guangdong China; 7 BGI-Tianjin, BGI-Shenzhen, Tianjin, China; 8 Department of Radiology, Beijing Tiantan Hospital, Capital Medical University, Beijing, China; 9 Tiantan Neuroimaging Center of Excellence, Beijing, China; 10 BGI-Shenzhen, Shenzhen, Guangdong China; 11 Guangdong Provincial Key Laboratory of Genome Read and Write, BGI-Shenzhen, Shenzhen, Guangdong China; 12 James D. Watson Institute of Genome Sciences, Hangzhou, Zhejiang China; 13 Department of Epidemiology and Biostatistics, Ministry of Education Key Laboratory of Environment and Health, State Key Laboratory of Environmental Health (Incubating), School of Public Health, Tongji Medical College, Huazhong University of Science and Technology, Wuhan, Hubei China

**Keywords:** Population genetics, Genome-wide association studies

## Abstract

Ischemic stroke is a leading cause of global mortality and long-term disability. However, there is a paucity of whole-genome sequencing studies on ischemic stroke, resulting in limited knowledge of the interplay between genomic and phenotypic variations among affected patients. Here, we outline the STROMICS design and present the first whole-genome analysis on ischemic stroke by deeply sequencing and analyzing 10,241 stroke patients from China. We identified 135.59 million variants, > 42% of which were novel. Notable disparities in allele frequency were observed between Chinese and other populations for 89 variants associated with stroke risk and 10 variants linked to response to stroke medications. We investigated the population structure of the participants, generating a map of genetic selection consisting of 31 adaptive signals. The adaption of the *MTHFR rs1801133-G* allele, which links to genetically evaluated VB9 (folate acid) in southern Chinese patients, suggests a gene-specific folate supplement strategy. Through genome-wide association analysis of 18 stroke-related traits, we discovered 10 novel genetic-phenotypic associations and extensive cross-trait pleiotropy at 6 lipid-trait loci of therapeutic relevance. Additionally, we found that the set of loss-of-function and cysteine-altering variants present in the causal gene *NOTCH3* for the autosomal dominant stroke disorder CADASIL displayed a broad neuro-imaging spectrum. These findings deepen our understanding of the relationship between the population and individual genetic layout and clinical phenotype among stroke patients, and provide a foundation for future efforts to utilize human genetic knowledge to investigate mechanisms underlying ischemic stroke outcomes, discover novel therapeutic targets, and advance precision medicine.

## Introduction

Stroke is the leading cause of mortality and long-term disability worldwide^1^. As a complex disease with diverse risk factors, clinical manifestation, and intermediate pathogenesis processes, the genetic heritability of stroke has been estimated to range from 16.1% to 40.3% according to small or mediate scale twin studies^2^ and genome-wide complex trait analysis^3^. Consequently, characterizing the relationship between sequence variation and rich stroke phenotypes becomes crucial in order to understand the mechanisms of stroke pathogenesis and prognosis, and to develop novel therapeutic strategies for disease prevention and treatment^4^. Efforts, such as MEGASTROKE^5^, have made some progress, revealing 32 stroke risk loci harboring common genetic variants. The recent GIGASTROKE study also reported 89 independent loci for stroke risk using the same meta-analysis strategy^6^. In addition, several studies have contributed to revealing genetic risk factors underlying post-stroke outcomes^7–10^. While those studies were mostly array-based and focused on European populations^5,11–15^, challenges remain in a better understanding of the genetic effects of the complete set of coding and noncoding variants across the allele frequency spectrum on well-defined intermediate phenotypes and clinical outcomes, and the impact of ancestral differences, as well as a systematic elucidation of the molecular mechanisms underlying the occurrence and progression of the disease^16^. Whole-genome sequencing (WGS) of a well-characterized patient registry with comprehensive medical records, and long-term follow-up on stroke outcomes among the underrepresented East Asian populations provides a foundation to address these challenges.

Launched in China, the STROMICS study represents the first and largest endeavor to understand how stroke occurs, recurs, repairs, and recovers using a multi-omics and systems biology strategy. Here, we outline the STROMICS design and resource and report findings from the STROMICS Phase I genome study, following a common thread to elucidate genetic characteristics and discoveries of a new genomic resource^17^. The major findings were achieved based on the deep WGS (on average 41.17×) and analysis of 10,241 patients recruited from the Third China National Stroke Registry (CNSR-III)^18^, a China nationwide prospective registry for patients presented to hospitals with acute ischaemic cerebrovascular events with long-term follow-up^19^. We constructed a high-quality variation dataset consisting of 135.59 million single-nucleotide variants (SNVs) and insertions and deletions (indels), including 42% novel variants not present in dbSNP (Build 155). We dissected the fine-scale genetic structure of the participants, reported the most comprehensive map to date of genetic loci under natural selection across the latitudinal and longitudinal gradients in China, and elaborated on how the knowledge of population genetic history may impact disease prevention and medication strategy. We further investigated the genetic-phenotypic associations of 18 stroke-related traits, including 14 biochemical indicators, 2 neuroimaging, and 2 behavioral traits, from both the common and rare variant perspectives. Finally, based on a complete variant call set of the *NOTCH3*, a causal gene for the autosomal dominant stroke disorder cerebral autosomal dominant arteriopathy with subcortical infarcts and leukoencephalopathy (CADASIL), we systematically analyzed the phenotypic spectrum of individuals carrying functional variants in the gene. The genomic resources and knowledge obtained from this study provide a robust foundation for further endeavors to investigate undermined mechanisms for stroke onset and progression, and expedite the exploration of new targets for stroke primary and secondary prevention. The STROMICS website is available at http://www.stromics.org.cn.

## Results

### The STROMICS design and resource

**Fig. 1:**
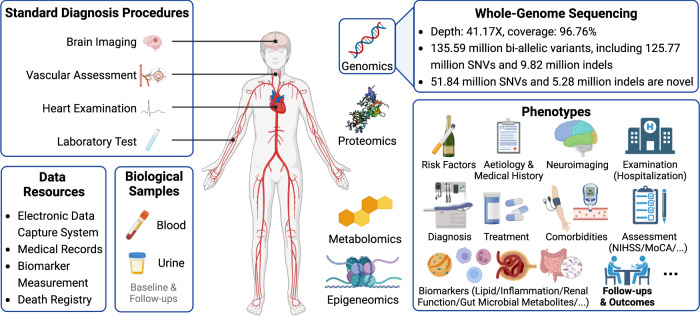
Summary of the major components of the STROMICS resource and WGS content. Individuals were recruited from the CNSR-III and underwent a series of standard diagnostic procedures according to the WHO criteria, and acute ischemic stroke was confirmed by MRI or brain CT. An electronic data capture (EDC) system was developed and used for data collection. Clinical phenotypes were extracted from EDC, medical records during hospitalization, biomarker measurement from biological samples, and death registry from the Chinese Center for Disease Control and Prevention (CDC). Individuals were followed up at 3 months, 6 months, and 1–5 years annually. Blood and urine samples were collected in face-to-face visits (baseline and follow-ups), and were stored in the Beijing Tiantan hospital. Omics screening is performed on the blood of the individuals. The number of high-quality genetic variants identified from the WGS is shown. Figure created with BioRender.com.

**Fig. 2:**
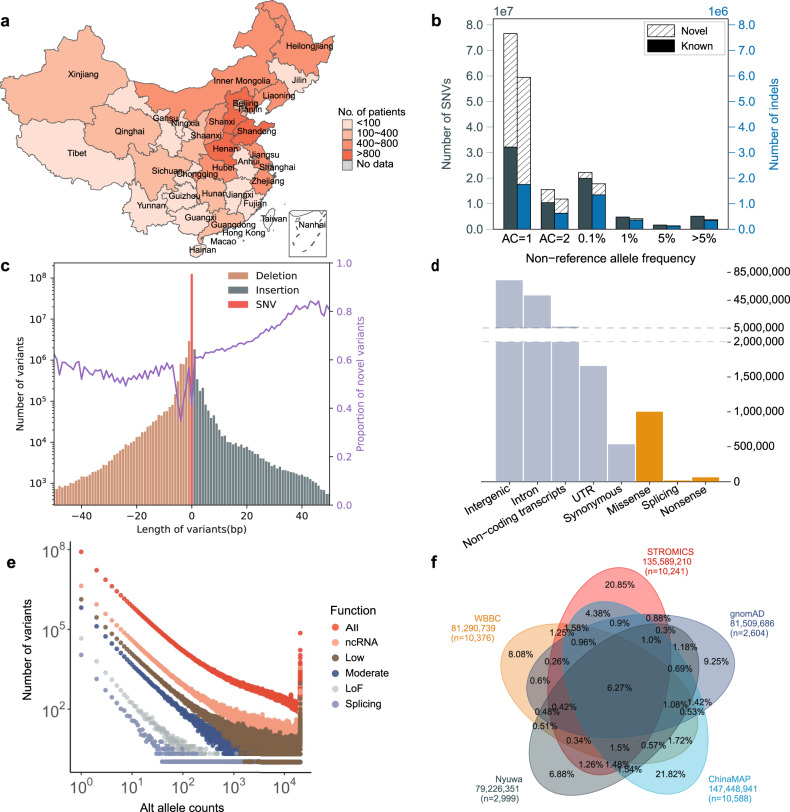
Allele frequency spectrum and functional annotation of the 135.59 million genetic variants among 10,241 individuals from STROMICS. **a** The geographical distribution of STROMICS samples in China. **b** The number and allele frequency spectrum of STROMICS variants (SNVs and indels). Novel and known variants are defined by dbSNP (Materials and methods). AC, allele count. **c** Length and number distribution of STROMICS variants. The purple line shows the proportion of novel variants. **d** The total number of variants observed in each functional class of genome. **e** Relationship between alternative allele count and the number of variants among different functional categories. The function categorization of the genetic variants (All, LoF, splicing, Moderate, Low, ncRNA) was shown in Supplementary Table S8. **f** Venn diagram showing the concordance of genetic variants among STROMICS, gnomAD, ChinaMAP, NyuWa Genome resource, and WBBC.

The STROMICS aims to characterize the mechanisms underlying the phenotypic spectrum of stroke patients before and after stroke onset, from the multilayer omics perspectives. The Phase I study focuses on the genome study of 10,241 unrelated patients from CNSR-III (Supplementary Fig. S1 and Table S1), a nationwide prospective registry targeting the prognostic outcome of patients presented to hospitals with acute ischaemic cerebrovascular events between August 2015 and March 2018 in China^18,19^. A wide variety of phenotypic information as well as biological samples (blood and urine) were collected from each participant (Fig. 1). Acute ischemic stroke was diagnosed according to the WHO criteria^20^ and confirmed by brain magnetic resonance imaging (MRI) or computed tomography (CT). The geographical distribution of birthplace among the 10,241 STROMICS participants covered 31 out of the 34 provincial administrative divisions of China (Fig. 2a; Supplementary Table S2).

The baseline data included prehospital care, pre-stroke modified Rankin Scale (mRS), National Institutes of Health Stroke Scale (NIHSS) score, age, blood pressure, clinical features, duration of symptoms, ABCD2 score, patient demographics, medical history, family history, previous medication, physical examination, primary diagnosis, laboratory tests, risk factor assessment, and brain imaging (including brain MRI or CT, and at least one intracranial vascular assessment). Etiology classification of ischemic stroke was performed according to the TOAST (Trial of Org 10172 in Acute Stroke Treatment) criteria^21^. At discharge, the research coordinators extracted the auxiliary examination and recorded standard etiological evaluation results, medication, vascular-related operation and surgical procedures, final diagnosis, NIHSS and mRS score, economic burden, and cerebrovascular events during hospitalization. Patients were followed up, interviewed face-to-face at 3 months, and contacted over the telephone by trained research coordinators at 6 months and 1–5 years annually. Information including functional status, cardiovascular/cerebrovascular events, compliance with recommended secondary prevention medication, and risk factor control was queried at each follow-up.

Blood and urine samples were collected within 24 h of admission, at 3 months, and 12 months. DNA was extracted from the white blood cells of the participants, and subjected to deep WGS using 100 bp paired-end reads to high sequencing depth (41.17× on average), covering ~96.76% ± 0.17% of the non-N sequence human genome with an average insert size of 236 bp (Materials and methods).

### 135.59 million high-quality genetic variants

After read alignment, variant detection, and a series of variant-level quality control (Materials and methods), we identified a call set of 135,589,210 biallelic variants, including 125,769,898 SNVs with Ts/Tv ratio of 2.06 (Fig. 2b; Supplementary Fig. S2), and 9,819,312 indels ranging from 1 bp to 50 bp in length (Fig. 2c; Supplementary Table S3). To assess the accuracy of the variant call set, we compared the genotypes of the union set of SNVs and indels in 50 individuals who were also sequenced to 198.03× in a gene panel (Materials and methods). The false discovery rate (FDR) for the identified SNV and indel was 1.56% and 1.12%, respectively, indicating robustness of the variants detected and genotyped in this study (Supplementary Table S4). To investigate the allele frequency spectrum of the 135.59 million variants, we categorized the variants into six groups based on the alternative allele frequency estimated from the 10,241 patients (Fig. 2b; Supplementary Table S5). The majority of the SNVs and indels were found to be rare, with a minor allele frequency (MAF) of < 0.1% (SNVs: 114.49 million, 91.03%; indels: 8.92 million, 90.86%; Supplementary Table S3). A total of 51.84 million (41.22%) SNVs (Ts/Tv = 1.36) and 5.28 million (53.79%) indels were not cataloged in dbSNP (Build 155), with most of them being rare variants (Fig. 2b; Supplementary Table S5).

The length distribution of the 1–50 bp indels was symmetric, with periodical peaks at 2 bp across the genome, corresponding to the polymerase slippage mechanism of indel generation. Additionally, peaks at 3 bp were observed specifically in the exonic regions, likely resulting from purifying selection acting on the frameshift indel in the coding region (Fig. 2c; Supplementary Fig. S3 and Table S6)^22^. While the majority of the 135.59 million genetic variants were located in the non-coding region (98.81%), 1.61 million variants (1.19%) were found in the coding region, including 534,090 synonymous SNVs, 1,001,334 missense, stop-loss, and non-frameshift variants, 14,123 splice-site, and 60,066 loss-of-function (LoF) variants (Fig. 2d; Supplementary Table S7). The variant allele frequency tended to decrease with the increasing deleterious consequences (Fig. 2e; Supplementary Table S8). Notably, we observed that the frequency of the splice-site variants declined faster than the LoF variants such as frameshift, stop-gain, start-loss, and initiator codon variants, suggesting stronger purifying selection against the splice-site variants.

Compared to all the other four WGS studies among Chinese and East Asian populations, including China Metabolic Analytics Project (ChinaMAP, v1.0; 40.8×; 10,588 genomes)^23^, Nyuwa (v1.0; 26.2×; 2999 genomes)^24^, Genome Aggregation Database (gnomAD) EAS (v3.1; 2604 genomes)^25^ and Westlake BioBank for Chinese (WBBC, v20211129; 13.9×; the combination of 4535 WGS genomes and 5841 arrays)^26^, the STROMICS genome study reported here has uniquely contributed 64.77 million (20.85%) from the union set of 310.72 million SNV and indel variants from the five studies (Fig. 2f). As a sanity check, we compared the allele and genotype frequencies of 89 genetic variants associated with stroke occurrence from the latest genome-wide association study (GWAS)^5,6^ as well as 10 variants associated with response to three drugs for stroke treatment (clopidogrel, warfarin, statin) between the STROMICS population and the other reference populations (Supplementary Tables S9 and S10). Six out of the 89 stroke risk variants were not detected in the STROMICS or were detected in less than two of the reference datasets and were therefore not discussed (Supplementary Table S9). The remaining 83 loci consisted of 58 variants associated with overall risk of stroke occurrence and 25 variants associated with the risk of specific subtypes, including the large artery stroke (LAS), cardioembolic stroke (CES), or small-vessel occlusion (SVS). We found that 37 out of the 83 variants associated with stroke risk showed significantly higher allele frequencies among STROMICS patients compared to at least two of the other four Chinese WGS reference datasets (χ^2^ or Fisher’s exact test *P* < 0.05). For the remaining 46 variants, 17 variants showed unexpectedly lower frequency in STROMICS compared to the reference populations (Supplementary Fig. S4), and 29 variants did not show a consistently significant difference. To understand whether the discrepancy is due to ancestral differences, we explored eight of the loci associated with stroke risk among the East Asian populations. We found clear evidence for stroke risk for four loci (*SH2B3*, *FGF5*, *PITX2*, and *KCNK3*) (Supplementary Fig. S5). However, the risk allele of the two loci (*COL4A2* and *SH3PXD2A*) showed lower frequencies in STROMICS compared to the reference populations. For two loci that were associated with stroke risk among the South Asians (SAS, *COBL*) and Africans (AFR, *PITCH1*), the *PTCH1* locus also showed unexpectedly lower frequency in STROMICS compared to the reference populations. Nonetheless, it is worth noting that the unexpected allele frequency difference observed between the STROMICS patients and the control datasets may be due to a lack of correction of population structure and other confounding factors in the analysis^27^. Future studies investigating the genetic determinants of stroke risk among the non-European underrepresented populations should prioritize leveraging the STROMICS Phase I resource as a high-quality patient dataset. For the 10 genetic variants associated with pharmacogenetics, one variant *rs12248560-T* in *CYP2C19* associated with a poorer response to the antiplatelet clopidogrel therapy consistently showed significantly higher allele frequency among STROMICS compared to the other reference populations (Supplementary Fig. S6 and Table S10).

Furthermore, we compared the genotype frequencies of the 99 genetic variants between STROMICS and 10 populations from the gnomAD consortium (Supplementary Tables S11, S12 and Figs. S7, S8). The STROMICS and the gnomAD East Asian populations share a higher proportion of stroke risk-associated variants with similar allele frequencies (61 out of the 83 variants, 73.49%, *P* > 5.62 × 10^–4^) compared to the other nine populations (Non-Finnish European ancestry: 9.64%; Finnish ancestry: 3.61%; South Asian ancestry: 14.46%; Middle Eastern ancestry: 28.92%; Ashkenazi Jewish ancestry: 16.87%; Latino ancestry: 8.43%; Amish ancestry: 16.87%; African/African-American ancestry: 3.61%; other ancestry: 15.66%; *P* > 5.62 × 10^–4^). As an example, the genotype frequency distributions of the 41 variants displaying the most significant discrepancy (*P* < 5.0 × 10^–324^) between the East Asian and the Non-Finnish European populations are shown in Supplementary Fig. S7. These discrepancies in genotype frequencies for the stroke risk-associated variants among various populations suggest the presence of a population-specific genetic basis for stroke risk. We can draw several conclusions from the genotype frequency distribution of 10 genetic variants related to drug response. First, statins, which are commonly prescribed for cholesterol reduction and stroke prevention, may have differing effects based on genetic profiles. The higher frequency of *rs4149056-C* in *SLCO1B1* and *rs2231142-T* in *ABCG2* among the Chinese and East Asian populations suggests an increased statin exposure and a higher risk of myopathy in statin users^28^. Therefore, clinical trials for statin therapies for stroke should take into consideration the population- and individual-specific genetic profile, which can benefit from the genomic resources provided by STROMICS. Second, for warfarin and clopidogrel, which are widely used as oral anticoagulants worldwide^29^, the higher frequency of the *VKORC1* (*rs9923231-T*), *CYP4F2* (*rs2108622-C*), *CYP2C9* (*rs1057910-C* and *rs1799853-T*), and *CYP2C18* (*rs12777823-A*) alleles associated with more sensitive response to warfarin^29^ and the *CYP2C19* (*rs4244285-A*, *rs4986893-A*, and *rs12248560-T*)^30^ alleles associated with poorer clopidogrel metabolism, suggest that a lower warfarin dose and an alternative antiplatelet agent other than clopidogrel should be considered among the Chinese and Asian populations.

### Population genetic structure and natural selection

Knowledge of the genetic structure and history provides essential information for disease study using association tests^31^ and has not yet been assessed among the STROMICS patients. We applied genetic methods including principal component analysis (PCA)^32^, ADMIXTURE^33^, and PC-based selection^34^ to quantify the population structure and identify recent positive selection using the WGS data from STROMICS.

**Fig. 3:**
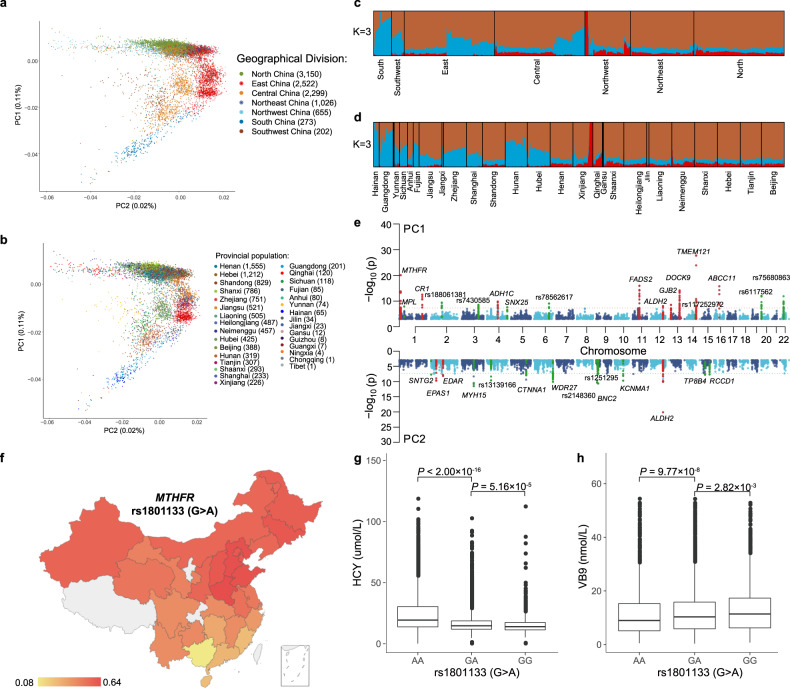
Population structure and adaptation. **a**, **b** PCA of all the individuals in STROMICS (*n* = 10,241) colored by seven geographical regions (**a**) and by 31 provincial divisions (**b**). Each point represents one participant and is placed according to their eigenvectors. **c**, **d** Distribution of the 3 ancestry components in STROMICS participants (*n* = 10,241) of geographical region (**c**) and of provincial divisions (**d**) as inferred using the ADMIXTURE for K = 3. Each color reflects one of the three ancestral components. The proportion of ancestral components for each individual was indicated by a stacked bar. Individuals were organized by provinces along the *x*-axis. **e** Genomic signatures under selection along PC1 (upper panel) and PC2 (lower panel). The nearest gene of the lead SNV for each selection signal is indicated. **f** A geographical distribution of the A allele frequency of the SNV *rs1801133* (chr1:11796321) in the *MTHFR* and *CLCN6* loci under genetic selection in the STROMICS population. Provinces with a sample size of < 5 were filled in gray. **g**, **h** Linear regression of homocysteine and VB9 (folate) on the three genotypes of *rs1801133*, respectively, with gender, age, history of stroke, and the day duration between stroke onset and the blood sampling as the covariates.

We revealed a fine-scale genetic structure that closely mirrored the geographical distribution of the patients using the PCA (Fig. 3a, b; Supplementary Fig. S9). The first two principal components, PC1 and PC2, explained a total of 0.13% variance and formed multiple clusters that corresponded to the seven geographic regions of China (Fig. 3a) and the 31 provincial administrative divisions (Fig. 3b). Further analysis using linear regression demonstrated that PC1 was associated with latitude (*r* = 0.74, *P* < 2.2 × 10^–16^), and PC2 was associated with longitude (*r* = 0.44, *P* < 2.2 × 10^–16^) (Supplementary Fig. S10). Notably, population stratification was more evident when including low-frequency variants (0.01 < MAF < 0.05) and rare variants (0.005 < MAF < 0.01) in addition to common variants (MAF > 0.05) in the PCA analysis (Supplementary Fig. S11). When comparing the STROMICS patients with 26 populations from the 1000 Genome Project Phase 3 (1KGP3) using PCA, as expected, the majority of STROMICS patients clustered with the 1KGP3 East Asian populations, while a small proportion of minorities showed evidence of ancient admixture with European or South Asian populations (Supplementary Fig. S12). We also employed ADMIXTURE with a model of 3 and 11 hypothetical ancestral components, selected based on the smallest cross-validation error, to analyze the STROMICS patients alone and in combination with the 26 populations from 1KGP3 (Supplementary Fig. S13a, b). Our results showed that the STROMICS Phase I patients were mainly composed of three ancestral components, representing northern and southern Chinese ancestries, as well as a component from the northwest that likely originated from ancient European gene flow (Fig. 3c, d). Compared to the CHB, CHS, and CDX Chinese populations from 1KGP3, the STROMICS patients showed a higher average percentage of ancestry from North China, compensating for the missing diversity in the 1KGP3 study (Supplementary Fig. S13c, d).

We subsequently investigated recent genetic adaptation by analyzing variants with unusual allele frequency distribution along each PC against a null model of genetic drift (See Materials and methods). We identified 17 and 14 loci that showed significant allele frequency differences across PC1 and PC2, respectively, which correspond to the latitudinal and longitudinal gradients on the geographical map of China (adjusted *P* < 2.5 × 10^–8^) (Fig. 3e; Supplementary Figs. S14 and S15). Among these loci, 12 gene loci (*MTHFR*, *CR1*, *EPAS1*, *EDAR*, *ADH1C*, *FADS2*, *ALDH2*, *GJB2*, *DOCK9*, *IGH* cluster, and *ABCC1)* were previously reported to be under selection in East Asians or other populations, and 19 were newly identified selection signals (Supplementary Tables S13 and S14).

Notably, we observed a strong selection signal at rs1801133, a coding single-nucleotide polymorphism (SNP) known as Ala222Val or C677T or G677A, in the methylenetetrahydrofolate reductase gene (*MTHFR*) on chromosome 1. The frequency of the A allele of rs1801133 demonstrated a latitude-dependent gradient trend across China, with the highest frequency observed around the latitude of 40 degrees North (Fig. 3f; Supplementary Fig. S16). This pattern of allele frequency distribution across the 34 administrative divisions across China has not been reported before but is consistent with previous findings across Eurasia^35,36^. The observed adaptation generally supports a model^35^ proposing that individuals with limited access to green vegetables and/or living in places with high UV radiation would benefit from the Ala variant (the C or G allele) for elevated folic acid synthesis, to compensate for an inadequate dietary intake or the UV photolysis influence on folate^36^. Despite the historic limitations in access to green vegetable supplements in North China, the high frequency of the A allele, rather than the more efficient G allele, among northern Chinese suggests a weaker effect of dietary supplies than UV radiation on folate deficiency.

We then combined the rich phenotypic information in the STROMICS to bridge the selection signal and potential medical relevance. We found that the G677A allele frequency was significantly associated with an increase in homocysteine (*P* = 5.94 × 10^–69^, Fig. 3g; Supplementary Fig. S17a, b) and a decrease in serum folate acid concentration (*P* = 5.82 × 10^–14^, Fig. 3h; Supplementary Fig. S17c) among the STROMICS patients. This was expected, as the G-to-A mutation results in an alteration of alanine to valine, leading to reduced MTHFR activity^37^. We also observed that the A allele showed a significantly higher frequency in the STROMICS compared with all four Chinese reference datasets, suggesting an association of the G677A mutation with increased stroke risk in the Chinese population without adjustment for population stratification (χ^2^ test, *P* < 3.63 × 10^–18^) (Supplementary Table S15). When we analyzed the summary statistics from the recent GIGASTROKE study on stroke risk, we confirmed that the association of stroke risk with the variant was present among East Asians but not in the European population (Supplementary Table S15), consistent with a previous meta-analysis reporting a higher odds ratio (OR) of G677A for stroke risk in populations with a low folate supplementation compared to populations with folate fortification^38^. This evidence suggests that folate nutrition is still insufficient among the Chinese population for stroke protection. A gene profile-based prevention strategy can be derived as follows: for populations in North China where a higher incidence rate of cardiovascular and cerebrovascular diseases has been observed^39^, regularly enhancing folate intake to lower homocysteine levels among individuals with *MTHFR 677A* allele and possibly other populations with similar genetic and environmental conditions may be an effective prevention strategy to reduce the disease burden. For populations in southern China where most individuals have *MTHFR 677G* allele, protection from high environmental UV radiation may be a more efficient method to reduce folate deficiency, especially considering that current folate intake is more intensive in southern provinces compared to northern provinces according to the China CDC^40^. Future clinical trials will be valuable for validating these predictions.

In addition to the *MTHFR* selection signal, we identified 16 additional genetic loci that showed evidence of selection along the latitude (Fig. 3e; Supplementary Fig. S14). Among these, nine loci have been previously reported to be under selection. For instance, the *CR1* and *TMEM121* loci have been shown to be adapted to pathogen infection in South China^41^. The *ADH1C* and *BRAP* (*ALDH2*) loci are known to be involved in alcohol metabolism and have been associated with agricultural development in East Asia^42–44^. The *MYRF* (*FADS2*) locus has been shown to be adapted to a diet lacking fatty acid in some populations in Eurasia^41,45^. The *GJB2* locus has been associated with recessively inherited forms of deafness and is thought to have been selected for potential advantage related to epidermal thickening or increased cell survival^46,47^. The *DOCK9* locus has been suggested to be adapted to potential advantages related to bone mineral density^41^, and the *ABCC1* locus has been associated with adaptation to cold climates in East Asians^41^ and other populations^48^. Selection signals along the China longitude were first reported in this study (Fig. 3e; Supplementary Fig. S15). Among the 14 significant signals identified, the strongest signal was observed for the *BRAP* (*ALDH2*) locus, suggesting that adaptation of the alcohol metabolism to agricultural development occurs along both the latitude and longitude in China. The other two selection signals with evidence from previous studies include the adaptation of erythrocyte abundance to hypoxia in high altitude (*EPAS1* locus), which was first discovered in the Tibetan population in northwest China^49^ and a proposed adaptation of mother-to-infant transmission of vitamin D and fatty acid through breast milk during the last ice age (*EDAR* locus)^50^. Six of the 19 newly identified signals have functions according to the GWAS catalog (Supplementary Tables S13 and S14): the *MPL* locus is associated with myelofibrosis and amegakaryocytic thrombocytopenia; the *SNX25* locus is associated with hair color measurement; the *SLC52A3-FAM110A* locus is associated with prostate cancer; the *TCN2* locus is associated with type 2 diabetes; the *TBC1D1-LINC01258* locus is associated with heel bone mineral density; and the *CTNNA1* locus was associated with susceptibility to digestive cancer. Interpretation of the selection signals as well as the 13 remaining signals without functional annotation, requires further phenomics information and basic experiments.

### Genome-wide associations of 18 stroke-related biochemical, behavioral, and imaging traits

A wide variety of physiological, biochemical, and behavioral risk factors were documented for patients recruited in the CNSR-III registry. The STROMICS genome study enables the discovery of the genetic determinants of numerous risk factors within a Chinese patient cohort, providing foundational knowledge for causal inference on stroke onset and outcome in future studies. We investigated a set of 18 representative traits related to stroke risk from both the common and rare variant perspectives which included 14 biochemical traits in four categories (lipid-related, homocysteine-related, inflammation, and kidney function-related biomarkers), two behavioral (drinking and smoking), and two imaging traits (diffusion-weighted imaging (DWI)-positive acute ischemic stroke and symptomatic extra- and intra-cranial atherosclerotic stenosis, abbreviated as AIS-DWI and sEICAS, respectively). GWAS studies have not yet been conducted among the East Asian populations for six of the traits (Apo-CII, Apo-CIII, Apo-E, PCSK9, AIS-DWI, and sEICAS). None of the 18 traits have been investigated among Chinese populations with WGS greater than 10 K individuals (Supplementary Table S16). Based on the power analysis, we were able to identify genetic variants with a power of 80% or more given its MAF ≥ 0.01 and an effect size of 0.47 or an OR of at least 2.57 providing the sample size in our study (Supplementary Fig. S18). In case of the presence of genetic variants with large effect sizes, we restricted the genome-wide association analyses for variants with MAF > 0.5% using linear mixed model in SAIGE^47^ (see Materials and methods). All analyzed traits had SNP heritability ranges from 1% to 16%, and GC lambda close to 1.0 (0.98 < λ_GC_ < 1.01), suggesting no significant inflation in association analysis (Supplementary Table S17).

**Fig. 4:**
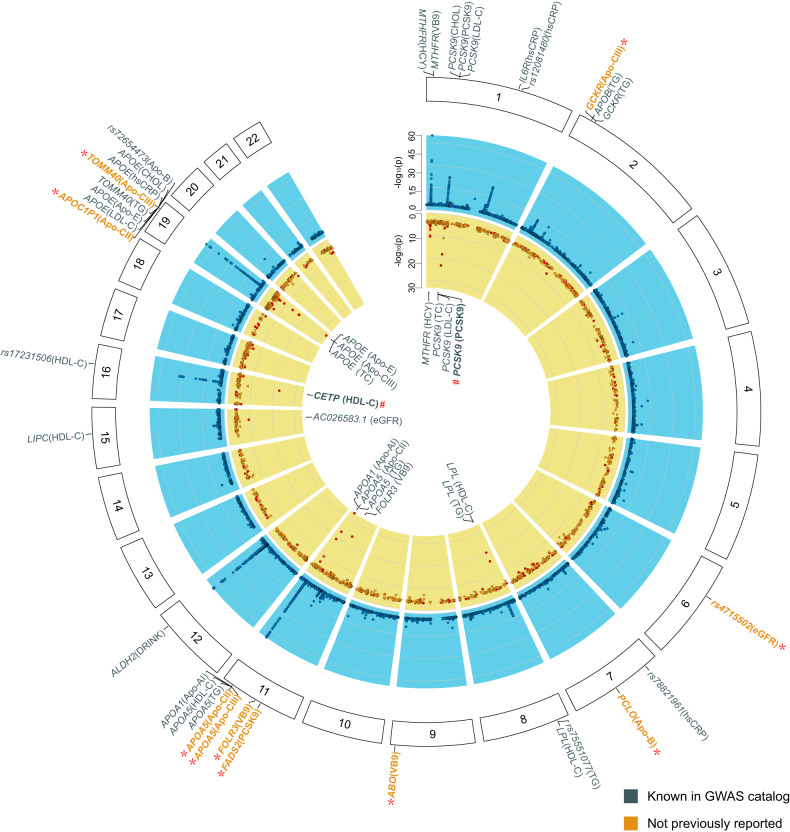
Circular presentation of single variant and gene-based association of stroke-related biochemical, behavioral, and imaging traits. Chromosomes were indicated by numbered panels 1–22. The –log_10_ *P* for single variant and gene-based genetic association with the traits by chromosomal position was shown by the blue and yellow panels, respectively. The significance threshold was *P* < 2.78 × 10^–9^ and *P* < 1.50 × 10^–7^ for single variant and gene-based association test correcting for the number of traits tested (*n* = 18) and the number of genes tested (*n* = 17,464). In the outermost blue panel, genetic loci, namely, the 1 Mbp window centering on the lead SNV, were indicated by the nearest gene of the independent lead SNV in the single variant association analysis. Genetic loci that had not been reported to be associated with the same trait in the GWAS catalog (v1.0.2-e105_r2021-12-21) were marked by a star (*) and shown in bold and orange. In the innermost yellow panel, gene loci that passed the gene-based association test using variants with MAF < 0.005 (Supplementary Table S20) are shown. Additionally, the *CETP* and *PCSK9* gene loci that also passed the gene-based association test using rare functional variants alone (Supplementary Table S21) were marked by number (#) and shown in bold. Color keys in the middle represent the categories of the traits.

In total, we identified 56 independent genetic associations reaching genome-wide significance for the 18 traits (*P* < 2.78 × 10^–9^), including 32 loci that were not previously reported for seven traits, such as Apo-B, Apo-CII, Apo-CIII, VB9 (folate acid), eGFR, and sEICAS (Fig. 4). Additionally, we summarized 21 genetic loci that reached genome-wide significance criteria (*P* < 5 × 10^–8^) in Supplementary Table S18. For 29 out of the 77 loci, we found replication data from the published GWAS analyses in the GWAS catalog^51^, Phennoscanner^52,53^, and OpenGWAS^54^ (see Materials and methods). All 29 loci were replicated based on the criteria of the same direction of effect and a significance level of *P* < 1.72 × 10^–3^ (Supplementary Table S18).

Among the 14 biochemical traits that include lipids (triglyceride TG and total cholesterol TC), lipoproteins (LDL-C and HDL-C), apolipoproteins (Apo-A, B, C, and E), the LDL receptor binding enzyme PCSK9, homocysteine (HCY), vitamin B9 (VB9), hypersensitive C-reactive protein (hsCRP), and estimated glomerular filtration rate (eGFR), we identified a total of 33 loci. Ten were first discovered in this study (*P* < 2.78 × 10^–9^) (Fig. 4; Supplementary Table S18). The 10 newly identified genetic associations consist of five loci for apolipoprotein CII and CIII of which GWAS studies were not conducted before, one locus tagged by an intronic indel at *PCLO* associated with Apo-B, one locus tagged by an intronic SNV at *FADS2* for PCSK9 (*rs651821*), one frameshift deletion at *FOLR3* (*rs71891516*), one locus tagged by an intronic SNV at *ABO* for VB9 (*rs9411377*) and one locus tagged by an intergenic SNV associated with eGFR (*rs4715502*). A few important discoveries can be summarized below. First, we found that although Apo-CII and Apo-CIII have different functions in the cholesterol metabolism pathway — Apo-CII activates LPL and processes chylomicrons and VLDL in circulation while Apo-CIII inhibits lipolysis by hindering the interaction of VLDL with the LPL complex^10^, their genetic determinants were similar (Supplementary Fig. S19). Both apolipoproteins were associated with variants near genes encoding APOC2 on chromosome 19, APOC3 on chromosome 11, and GCKR on chromosome 2, suggesting a close biological relationship between the two proteins. Secondly, we found that *FADS2* locus was the second most significant loci following the *PCSK9* locus that contributed to the plasma PCSK9 protein level among STROMICS Phase I participants. This locus was not previously found likely due to smaller sample sizes (*n* < 3290) in four published GWASs^51^. The intronic lead SNP *rs651821* was an expression quantitative trait locus (eQTL) for *FADS2* and its G allele increases both mRNA expression of *FADS2* according to GTEx^55^ (beta = 0.76, *P* = 2.1 × 10^–60^) and protein level of PCSK9 in the whole blood according to STROMICS (beta = 0.10, s.e. = 0.02, MAF = 0.34, *P* = 5.99 × 10^–11^). It is possible that the fatty acid desaturase 2 (FADS2), which plays a role in the synthesis of polyunsaturated fatty acids (PUFAs), may also play a role in cholesterol metabolism pathway. Further experiments are warranted to evaluate whether sharing of the genetic association between *FADS2* and *PCSK9* was attributed to causality or pleiotropy. Thirdly, we identified that a 2 bp frameshift deletion at *FOLR3* was the most significant variant that decreases folate level (*rs71891516-C*, beta = –0.25, s.e. = 0.03, MAF = 0.08, *P* = 1.58 × 10^–19^). The folate receptor gamma (FOLR3) was known to play a role in folate metabolism^56^. However, six GWAS studies on folic acid measurements did not find this association in either European or East Asian populations^51^. As *rs71891516* was not tagged well by nearby variants (the largest linkage disequilibrium (LD) *r*^*2*^ in a 1 Mbp window is 0.496 according to LDlink with the 1KGP3 populations as the reference panel), we infer that this new discovery benefits from the WGS design in STROMICS. The interpretation of the rest of the three newly identified loci was not immediate as the lead variants were not eQTLs according to GTEx portal^55^. Future replication and validation studies are required to understand these genetic associations.

We noted that extensive pleiotropy was involved in the genetic architecture of biochemical traits, especially lipid-related traits. Based on co-localization analysis for each significant locus between any two biochemical traits (see Materials and methods), we found that six loci around the *PCSK9*, *GCKR*, *ApoB*, *LPL*, *ApoC*, and *ApoE* genes on chromosomes 1, 2, 8, 11, and 19 showed shared genetic effect on at least two lipid traits (H4 > 0.8, Fig. 4; Supplementary Fig. S20 and Table S19). For example, *rs151193009* is a missense variant in *PCSK9* that simultaneously impacts levels of Apo-B, TC, and LDL-C (Supplementary Fig. S20a). *rs13306194* is a missense variant in *APOB* that shows shared genetic associations with Apo-CIII and TG (Supplementary Fig. S20b). *rs6547692* is an intronic variant in *GCKR* that influences levels of apo-CII, apo-CIII, and TG (Supplementary Fig. S20c). *rs75551077* is an intergenic variant around LPL that affects both HDL-C and TG (Supplementary Fig. S20d). A variant present in 5′ untranslated region (UTR) of *APOA5* affects Apo-CII, Apo-CIII, Apo-E, HDL-C, and TG (Supplementary Fig. S20e). *rs7412* is a missense variant that influences Apo-II, Apo-CIII, Apo-E, TC, LDL-C, and TG (Supplementary Fig. S20f). As molecules that partake in lipid metabolism have been regarded as well-established pharmaceutical targets for cerebrovascular disease^57^, sharing of genetic effects observed in STROMICS underscores essentiality of careful examination of horizontal pleiotropy in mendelian randomization analysis to discriminate true causal risk factors from artifacts.

For the two behavioral traits, we found that a long region (~2 Mbp) crossing the *ALDH2* gene on chromosome 12 (lead SNP *rs671*) was strongly associated with heavy drinking behavior (> 20 g/day) among the stroke patients (*rs671-G*, OR = 3.18, 95% CI = 2.83–3.59, MAF = 0.16, *P* = 2.43 × 10^–83^) (Fig. 4; Supplementary Figs. S19, S21 and Table S18). However, we did not find any significant genetic signals present in the *ADH1B* gene, which suggests that the drinking behavior among the STROMICS participants was mainly genetically driven by the *ALDH2* gene. We did not identify genetic associations with current smokers in the STROMICS Phase I patients, which was reasonable as known smoking genetic variants in East Asians have an OR between 1.1 and 1.3 and a MAF > 0.1^58^ and our study did not have enough power for discoveries (Supplementary Fig. S18d).

We identified 22 strong association signals affecting the risk of developing sEICAS for the two imaging traits (*P* < 2.78 × 10^–9^), which have not been investigated before in terms of genetic factors. The most significant gene locus on chromosome 20 increases at least three times the risk of developing sEICAS (OR 95% CI = 3.0–4.0). Almost all loci were present in the intergenic region except for two intronic SNVs: rs200364330 at *PDE5A* which regulates intracellular concentrations of cyclic nucleotides and plays a role in smooth muscle relaxation in the cardiovascular system^56^ and rs938277067 at *ZNF804A*, which was associated with schizophrenia and bipolar disorders^56^. Although no statistical inflation was observed (Supplementary Fig. S19 and Table S17) and the identified genetic associations met the power calculation (Supplementary Fig. S18), we believe that those association signals were likely false positives due to a lack of robust functional evidence from GTEx^55^ and Phennoscanner^52,53^ and a low MAF (< 0.05) observed for the lead variants. When all 210 significant principal components were included in the analysis, the results remained consistent with those based on the top five principal components. Therefore, false signals are likely to occur among low-frequency variants, even when using state-of-the-art GWAS algorithms with careful control of population stratification. Further investigation on the genetic associations between a small proportion of patients who were DWI-negative (1121 out of 8861, 12.65%) and the majority who were DWI-positive revealed an association signal tagged by a common intergenic variant (*rs6844814-G*, OR = 0.73, 95% CI = 0.66–0.81, MAF = 0.23, *P* = 1.95 × 10^–8^) that acted as an eQTL for *MFAP3L* in skin (*rs6844814-G*, beta = 0.12, *P* = 1.8 × 10^–4^). Replication of this signal in multi-ethnic populations will be warranted, considering that the DWI phenotype is easily available in stroke units.

We further investigated the potential genetic influence of the low-frequency and rare variants (MAF < 0.005) as well as those present in the coding sequence for the 18 traits using gene-based association tests, including SKAT-O, SKAT, and burden tests^59^. In the analysis of variants with MAF < 0.005, we identified 9 genes that exhibited distinct genetic burdens for 11 traits (*P* < 1.5 × 10^–7^) (Fig. 4; Supplementary Table S20). Notably, 8 out of the 9 genes, namely *MTHFR* for HCY, *PCSK9* for TC, LDL-C and PCSK9, *LPL* for HDL-C and TG, *APOA1* for Apo-AI, *APOA5* for Apo-CII and TG, *FOLR3* for VB9*, CETP* for HDL-C and *APOE* for Apo-CII, TC and Apo-E, overlapped with the genetic loci that contain common variant association signals. When we restricted the analysis to low-frequency and rare variants in the coding sequence, the most significant genes are *CETP* for HDL and *PCSK9* for PCSK9 (*P* < 1.5 × 10^–7^) (Supplementary Table S21). The consistent genetic associations observed for 8 out of the 9 genes in both common and rare variants in the non-coding sequence, compared to fewer genes found when restricting the analysis to variants in the coding sequence, suggest that non-coding variants may also play a causal role in the traits investigated in this study.

### Rare and functional *NOTCH3* variants implicate a broad phenotype spectrum

The extensive phenotypic data gathered in the STROMICS study also provides an opportunity to investigate the degree of penetrance between functional genetic variants and phenotypes in a clinical context. To illustrate this concept, we analyzed the phenotypic spectrum associated with all the functional variants in the causal gene *NOTCH3* for CADASIL, the most prevalent Mendelian stroke disorder characterized by autosomal dominant inheritance.

**Fig. 5:**
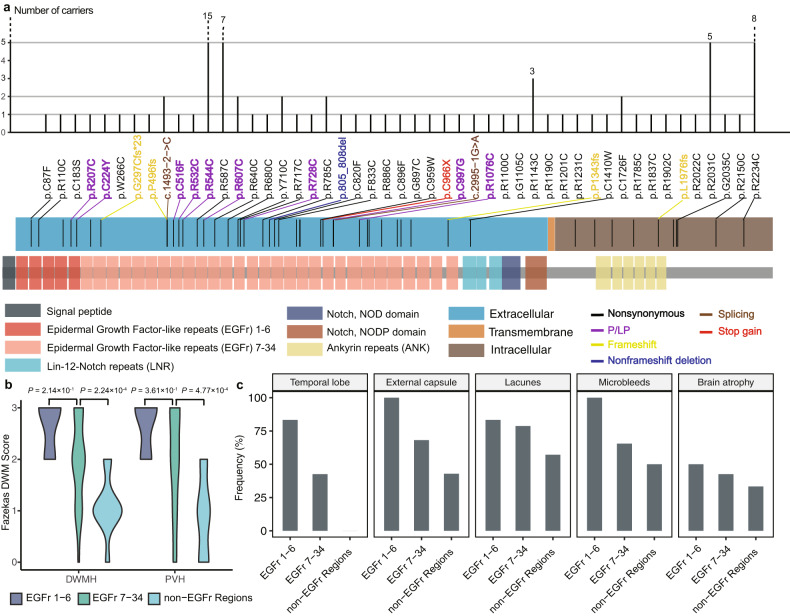
CADASIL-susceptible variants in STROMICS and neuroimaging of the carriers. **a** Distribution and number of carriers of CADASIL-susceptible variants in NOTCH3 protein. The variants are represented by their effect on amino acid in protein sequence according to Human Genome Variant Society Nomenclature. The upper panel shows the number of carriers for each variant. The variants are colored by their functional categorization. The lower panel shows the location of variants in NOTCH3 protein domains. **b** Violin plots of DWMH and PVH scores among carriers of Cys-altering SNVs in EGFr 1–6, 7–34, and non-EGFr regions. **c** Frequency of hyperintensities involving temporal lobe and external capsule, lacunes, microbleeds, and brain atrophy among carriers of Cys-altering SNV in EGFr 1–6, 7–34, and non-EGFr regions.

In the initial step, we conducted a review of the ClinVar database to obtain 4259 variants classified as pathogenic/likely pathogenic (P/LP) from the WGS call set (Supplementary Fig. S22a and Table S22). Among these, a total of nine P/LP variants (24 carriers) were identified in the *NOTCH3*. These variants all resulted in nonsynonymous substitutions between cysteine and other amino acids (Supplementary Table S23). Not considering ClinVar annotation, we identified 304 LoF, missense, non-frameshift indel, and splicing-site variants in total including 89 novel ones that were not documented in the dbSNP (Build 155) (Supplementary Fig. S22b). Since alterations in cysteine residues are considered significant characteristics of causal pathogenic variants in CADASIL^60^, we further extracted all nonsynonymous SNVs that resulted in Cys-altering changes (41 SNVs among 78 carriers, including the 24 carriers mentioned earlier), non-frameshift indels leading to loss or gain of Cys (1 deletion in 1 carrier), as well as 7 LoF variants including splice site variants (1 indel with 2 carriers and 1 SNV with 1 carrier), frameshift indels (4 indels with 4 carriers), and stop-gain SNVs (1 SNV in 1 carrier). Collectively, these 49 variants were designated as CADASIL-susceptible variants, and we further investigated the clinical characteristics of the 87 carriers in the subsequent analysis (Fig. 5a; Supplementary Tables S23–S25).

Given that CADASIL-susceptible variants may exhibit varying levels of deleteriousness in different domains of *NOTCH3*^61,62^, we categorized the 78 carriers (with 4 of them lacking MRI data) of Cys-altering SNVs into three groups based on the location of the variant within the epidermal growth factor-like repeat (EGFr) domain, and examined the brain MRI characteristics of these carriers (Fig. 5b, c; Supplementary Tables S26 and S27). In comparison to the 47 carriers of Cys-altering SNVs in EGFr 7–34 (amino acids 274–1373), the 6 carriers of Cys-altering SNVs in EGFr 1–6 (amino acids 40–272) exhibited more severe cerebral small vessel disease (CSVD) burden, including increased load of deep white matter hyperintensity (DWMH) lesions, periventricular hyperintensity (PVH) lesions, hyperintensity in the temporal lobe and external capsule, lacunes, microbleeds, and brain atrophy. Additionally, these two groups exhibited a heavier CSVD burden compared to the group of 21 carriers of Cys-altering SNVs in non-EGFr regions. These findings confirm that the clinical outcomes of the same type of variants are influenced by the sequence context.

To investigate the clinical heterogeneity among carriers of the same CADASIL-susceptible variant, the most frequently occurring variant resulting in p.R544C substitution in the NOTCH3 protein was analyzed. The impact of p.R544C on the structure of NOTCH3 protein was predicted using AlphaFold2^63^, which showed that the substitution of arginine with cysteine at amino acid 544 interferes with the ionic bond formed between p.R544 and p.E538, as compared to the wild type (Supplementary Fig. S23). Among the 15 carriers analyzed, varying degrees of white matter hyperintensity (WMH) load were observed, ranging from mild to severe (Materials and methods; Supplementary Fig. S24). There was a trend suggesting that the severity of WMH load was positively correlated with age, rather than other risk factors such as smoking status, drinking status, stroke history, hypertension, diabetes, and family history of stroke (Supplementary Table S28). However, none of these factors showed a significant correlation with the severity of WMH load. Since the p.R544C carriers did not carry additional CADASIL-susceptible variants, it is likely that the changing severity of WMH load is not due to additive genetic effects from variants in *NOTCH3*.

To explore the clinical features of carriers with other types of CADASIL-susceptible variants, we analyzed the remaining 8 variants (9 carriers), including the 7 LoF variants and 1 non-frameshift indel. All of the LoF variants, including 4 frameshift variants and 1 stop-gain variant, resulted in truncated proteins (Supplementary Fig. S25). Interestingly, among these carriers, only the individual with the stop-gain variant (p.C966X) exhibited a heavy CSVD load. In contrast, the remaining 8 carriers, despite carrying validated splice-site or frameshift LoF variants (see Materials and methods), showed a lower burden of CSVD compared to carriers of Cys-altering SNV carriers (Supplementary Fig. S24 and Table S25).

Finally, we made a comparison between the distribution of CADASIL-susceptible variants among STROMICS, the UK Biobank, and four Chinese non-stroke populations (Supplementary Table S23). The distribution of these variants showed substantial differences compared to the findings reported in the UK Biobank study^64^. The most common deleterious variant (p.R544C) in STROMICS was not detected in any of the 454,756 participants of the UK Biobank. Conversely, the most common Cys-altering *NOTCH3* variant (p.R1231C) identified in the UK Biobank study was found in only one patient in STROMICS. In the four Chinese or East Asian non-stroke populations (ChinaMAP, Nyuwa, WBBC, and gnomAD)^23–26^, a total of 67 carriers with 16 CADASIL-susceptible variants were identified. The frequency of carriers with CADASIL-susceptible variants was more than three times higher in stroke patients compared to non-stroke populations in China and East Asia (87/10,241 in STROMICS and 67/26,567 in ChinaMAP, Nyuwa, WBBC, and gnomAD).

## Discussion

The STROMICS genome study presented here is the first whole-genome study targeting an ischemic stroke patient registry. Through deep sequencing and analysis of a cohort of 10,241 patients from CNSR-III, we have identified a total of 135.59 million genetic variants. Notably, 57.12 million of these variants are novel, not previously cataloged in dbSNP (Build 155), and 64.77 million are SNVs and indels that have not been detected in other genome projects conducted in Chinese and East Asian populations. Our investigation of the allele frequencies of 89 stroke-associated genetic variants also revealed unexpectedly lower frequencies of 17 of these variants among the STROMICS patients, as compared to four Chinese reference datasets. However, as no correction of population stratification was applied, this finding requires further verification. Additionally, we observed significant differences in genotype frequencies of 41 genetic variants between STROMICS Chinese patients and European populations, highlighting the importance of considering ancestral differences when utilizing current genetic associations for stroke risk to develop novel therapies for stroke prevention and treatment in underrepresented populations. Analysis of the 10 genetic variants associated with response to three common drugs for stroke treatment also suggests potential benefits in utilizing genetic profile-based prescription and drug recommendations for stroke treatment.

Utilizing the common and the low-frequency genetic variants detected from the WGS dataset, we have dissected the population structure of the 10,241 patients and constructed a map of 31 genetic selection signals across the latitudinal and longitudinal gradients in China, including 12 signals that had been reported in separate studies previously. Notably, we identified the MTHFR-c.C677T (G>A, Ala222Val) variant as one of the most significant signals of adaptation. This variant was associated with increased homocysteine concentration, decreased folate levels, and increased risk for stroke, and was found to be enriched in the northern Chinese population. In contrast, the wild-type variant (677C or 677G or 222Ala) was more common in the southern Chinese population, likely due to adaptation to stronger UV radiation in southern China. Such genetic predisposition resulting from historical adaptation to the environment suggests that fortification of folate in dietary or medical intake is necessary for individuals in northern China, while protection from high environmental UV radiation is a more effective approach to reducing folate deficiency among those in southern China.

In our GWAS analysis of 18 stroke-related biochemical, behavioral, and imaging traits, we identified 56 independent genetic loci that reach genome-wide significance after Bonferroni correction (*P* < 2.78 × 10^–9^) and 77 reaching a genome-wide significance level (*P* < 5 × 10^–8^). We compared 29 out of the 77 loci with public GWAS summary statistics data and found that all loci were replicated. However, we cautiously interpreted the results of 22 loci associated with sEICAS (*P* < 2.78 × 10^–9^) as likely false positives based on functional annotation and allele frequency spectrum analysis. After excluding the sEICAS hits, we identified a total of 34 loci, including 10 newly identified loci for 6 traits that were first reported in this study. These novel loci included five for Apo-CII and Apo-CIII, for which the genetic basis had not been investigated before, one tagged by an indel variant in the intron of *PCLO* for Apo-B, one locus at *FADS2* for PCSK9, one locus at *FOLR3* for VB9 and one intergenic locus for eGFR. We found that the genetic determinants of Apo-CII and Apo-CIII were similar although the two apolipoproteins function differently in the cholesterol metabolism pathway. The novel association of the *FADS2* locus with plasma PCSK9 protein level and that of the *FOLR3* locus with folate level were functionally plausible, but had not been previously reported likely due to small sample sizes in previous studies. Although STROMICS has provided the most powerful knowledge on the genetic determinants of those traits and no statistic inflation was observed, replication and validation are necessary to understand the mechanism of the newly identified associations in the future. For the replicated loci, we also noticed a discrepancy in effect size for some of the loci between STROMICS patients and other cohorts (Supplementary Table S18). We infer that this discrepancy could be attributed to subtle ancestral differences or the physiological status of the patients. However, as no GWAS summary replication data from the Chinese population for the same biomarkers were publicly available, we are unable to distinguish the two different sources at the moment. We have made all the GWAS summary statistic data publicly available from STROMICS, and it will be interesting to investigate whether and how the patient status may change the genetic effect in the future. Notably, we identified extensive pleiotropy for six loci around the *PCSK9*, *GCKR*, *ApoB*, *LPL*, *ApoC*, and *ApoE* genes on chromosomes 1, 2, 8, 11, and 19. Each of these loci significantly impacted more than two biomarkers involved in lipid metabolism, raising caution for using Mendelian randomization to investigate the causal effects of the biomarkers for stroke outcomes, as horizontal pleiotropy may result in false interpretation. Interestingly, regardless of the functionality of the variants, loci defined by the rare variants called directly from the WGS data overlap with those defined by the common variants. Therefore, we infer that rare non-coding variants may also contribute to the phenotypic distribution.

Based on the data obtained from WGS, comprehensive medical records, and digital information, the STROMICS presents a unique opportunity to systemically investigate the genetic and neuroimaging landscape of ischemic stroke patients who carry CADASIL-susceptible variants. Previous understanding of CADASIL has suggested that it is an autosomal dominant condition, and individuals with *NOTCH3* variants that alter cysteine residues have been reported to exhibit a high burden of WMH on brain MRI, even in those who are not yet clinically symptomatic or manifesting symptoms^65^. However, our study has revealed that these *NOTCH3* variants were associated with a broad phenotypic spectrum in terms of WMH burden. In fact, only slight neuroimaging abnormalities were observed for 81-year-old patients carrying CADASIL-susceptible variants. Importantly, the severity of the CSVD burden is closely related to the location of the Cys-altering *NOTCH3* variants. In addition, among carriers with the LoF and splicing variants, only the patient with stop-gain mutation (c.C2898A; p.C966X) exhibited the severe form of CADASIL. The broad phenotypic spectrum observed in individuals carrying functional variants in the *NOTCH3* gene highlights the complexity of genetic and phenotype correlation in CADASIL. Moreover, carriers of CADASIL-susceptible variants may have a higher propensity to develop cerebrovascular diseases, and the distribution of *NOTCH3* deleterious variants in the Chinese population differs significantly from what has been reported in the European population.

Stroke is the second leading cause of mortality in the world and the leading cause of death in China, where a fifth of the world’s population resides^66–68^. Despite the passage of more than 80 years since the first identification of the connection between carotid artery occlusion and stroke in 1938^69^, it remains a significant global health issue with substantial social and economic implications. The history of stroke research serves as a model for investigating complex diseases. Unlike microarray genotyping, WGS is a rapidly advancing and relatively new technology for studying patients’ genomes at a population level. Genomic studies utilizing high-quality disease registry with comprehensive medical records and digital data bridges the patients’ genome and phenome. The findings from such a study enhance our understanding of the impacts of how population and individual genetic profiles impact intermediate molecular profiles and clinical outcomes. Future endeavors following this study will involve defining appropriate phenotypes and constructing a phenome-wide association map for more clinical and the multi-omics phenotypes collected or being collected in the STROMICS. Additionally, intensive efforts will be made to investigate the causal impact of the phenome on stroke outcomes and elucidate and validate the underlying mechanisms. Building on the genome study presented here, these efforts will facilitate the translation of genetic and molecular discoveries from STROMICS to effective therapeutics.

## Materials and methods

### Sample collection and study design

DNA samples were obtained from the CNSR-III^19^, which was a nationwide prospective registry for patients presented to hospitals with acute ischemic cerebrovascular events between August 2015 and March 2018 in China. The CNSR-III involved 201 hospitals that cover 31 out of the 34 provincial administrative divisions in China, including 163 grade III (central hospitals for certain districts or cities, usually teaching hospitals) and 38 grade II (hospitals serving several communities) urban hospitals. The written informed consent was obtained from all patients or legally authorized representatives before entering the study. Province of origin was extracted for each patient from the EDC system of CNSR-III.

### Sequencing and WGS data quality control

There is a total of 15,166 patients in CNSR-III cohort, and 10,914 patients in the prespecified genetic substudy were applied in WGS^18^. Library construction and WGS were conducted at BGI Genomics (BGI-Shenzhen) as previously described^18^. The WGS data were then processed under the Genome Analysis Toolkit (GATK) best practice guidance using Sentieon (release 201808.05)^70^. All of the reads were mapped to the non-N reference sequence of genome build GRCh38. Genetic variants in segmental duplications and unassigned chromosomes were excluded from analyses. Duplicated reads were removed, and base quality score recalibration was conducted.

Data quality control was performed by applying the filters listed below:

1. failed in library construction (*n* = 11);
2. microbial contamination (GC content ≥ 45%, *n* = 159);
3. 10× coverage < 80% (*n* = 15);
4. mismatch rate > 0.9% (*n* = 1);
5. FreeMix alpha > 0.03 (*n* = 267), which was calculated by VerifybamID2 to evaluate contamination from human DNA^71^;
6. genotype consistency rate < 85% (*n* = 4) for the 21 common SNVs that were used as a fingerprint for each patient and were independently genotyped using MassARRAY Spectrometry (Agena Bioscience, CA, USA).

A sex check of the data was performed using the depths of sex chromosomes. For each patient, the depths of sex chromosomes were normalized by the depth of the whole genome and were projected to a two-dimension plot, with coordinates of two axes indicating the normalized chrX depth and normalized chrY depth, respectively. After labeling each sample according to the reported gender, a margin naturally occurred (Supplementary Fig. S2c), and a simple horizontal line of normalized chrY depth of 0.075 was able to separate the male patients from the female patients, and thus was chosen as the threshold. Afterward, samples with inconsistency between inferred sex and recorded gender (*n* = 154), suspected sex chromosome aneuploidy (*n* = 11), or abnormal X chromosome heterozygosity (*n* = 13, outlier of the main clusters formed by male and female on the two-dimension plot) were excluded. The inferred sex for each patient was applied in further analyses.

Among the remaining patients, kinship relationship was inferred using KING v.2.1.8 software^72^, and 38 patients who had PI_HAT > 0.125 (indicating first- and second-degree relationship) with other patients were excluded.

Finally, a total of 10,241 genetically independent WGS data passed the quality control and were involved in further analyses (Supplementary Fig. S1).

### Variant calling and genotype quality control

Haplotyper of Sentieon was applied to call SNVs and indels for each individual. Then joint calling was performed after single-sample GVCF file was generated. Variant Quality Score Recalibration (VQSR) was performed for autosomes and sex chromosomes using GATK. First, variants with excessively heterozygous (ExcessHet > 54.69) were marked according to GATK best practice^73^. Then, the GATK bundle resource was used as known sites for the training step. The VQSLOD value was calculated for each genetic variant with the annotation including DP, QD, ReadPosRankSum, MQRankSum, FS, and SOR. The truth sensitivity levels were set at 99.0 and 98.0 for SNVs and indels, respectively (Supplementary Fig. S2a, b). Finally, variants that pass the VQSR filtration and with a QUAL > 30 were included for downstream analyses.

After VQSR, only biallelic SNVs and indels were retained for further analysis. The indels with length > 50 bp were excluded.

For each variant, the genotype for a patient was qualified if the depth (DP) was ≥ 9 and genotype quality (GQ) was ≥ 20. For heterozygous variants, allele depth (AD) should be ≥ 3. Otherwise, the genotype was set to missing for the corresponding patient. Genetic variants with call rate (patients with qualified genotypes/10,241) ≥ 85% were applied in this study.

We also divided the sex chromosome into pseudo-autosome regions (PAR), X-unique regions, and Y-unique regions according to the guidance in https://asia.ensembl.org/info/genome/genebuild/human_PARS.html. In PAR, the genotypes were extracted only from X chromosome and were applied in further analysis. Heterozygous variants in X- and Y-unique regions of male patients were set to missing genotypes. The call rates for X- and Y-unique regions were calculated by dividing chromosomes with qualified genotypes by the total number of chromosomes. For example, the call rate of qualified genetic variants in X-unique regions should be ≥ 85% in males (*n* = 7044, 7044 chromosomes), females (*n* = 3197, 6394 chromosomes), and the whole population (*n* = 10,241, 13,438 chromosomes) simultaneously.

The 135,589,210 qualified genetic variants after these procedures constituted the eventual STROMICS Phase I variation dataset.

### Variant annotation

All of the analyses in this study were conducted using the SNVs and indels with alternative allele frequency > 0. The function of these genetic variants was annotated by ANNOVAR with RefGene definition^74^. LoF variants included frameshift, stop-gain, start lost, and initiator codon variant (resulting in nonsynonymous amino acid substitution or deletion of the translation initiation codon in the gene). Splicing-site variants referred to the SNVs and indels at classical ±1 and ±2 splicing sites in protein-coding genes. The potential to induce alternative splicing was predicted using spliceAI for the splicing-site variants^75^. Variants in non-coding RNA (ncRNA) meant that the genetic variant was mapped to exonic, intronic, or UTRs of an ncRNA or non-coding transcript.

To explore whether the SNVs and indels in the STROMICS dataset had been reported or not, we downloaded the dbSNP database (https://ftp.ncbi.nih.gov/snp/.redesign/.archive/b155/VCF/GCF_000001405.39.gz). A genetic variant in STROMICS dataset would be regarded as a reported variant only if it had the same genomic coordinate, reference allele, and alternative allele with a variant that was deposited in dbSNP. Otherwise, the genetic variant would be regarded as a novel variant.

### Evaluation of the accuracy of WGS by comparison with target-capture next-generation sequencing (NGS)

Among the 10,241 qualified WGS samples, target-capture NGS was conducted for 50 randomly selected patients to evaluate the credibility of the WGS data. The target-capture panel was designed previously^76^. The panel covered exons of 446 genes that were implicated in hereditary cerebrovascular disease. DNA libraries were prepared using KAPA Library Preparation Kit (Kapa Biosystems, KR0453, Wilmington, MA, USA) following the manufacturer’s instructions. Paired-end reads (150 bp) were obtained from the Novaseq platform (Illumina, San Diego, CA, USA). The average depth for the covered regions in autosomes was 196.72× for the 50 patients. To construct the truth dataset, we generated VCF and GVCF files for each of the 50 patients using the targe-capture NGS data of the panel. Joint calling was conducted for all of the autosomal genetic variants. Both the consolidated GVCF files of joint calling and individual VCF files for each patient were subjected to the identical hard filters by which SNVs with QD < 2.0, FS > 60.0, MQ < 40.0, MQRankSum < –12.5, or ReadPosRankSum < –8.0, and indels with QD < 2.0, FS > 200.0, or ReadPosRankSum < –20.0 were eliminated. All of the multiallelic variants were eliminated, and genetic variants with GQ < 20, DP < 9, or heterozygous variants with AD < 3 were also eliminated. Indels with a length > 50 bp were excluded. Truth data were obtained after all of the above filtration procedures.

The query data were constructed from the WGS dataset after VQSR. For the identical 50 patients, we extracted all of the autosomal genetic variants in the target regions that were covered by the panel. Then, multiallelic variants, as well as variants with GQ < 20, DP < 9, and heterozygous variants with AD < 3 were also eliminated. The query data from WGS were compared with truth data using Hap.py (https://github.com/Illumina/hap.py). The number of true positive and false positive variants was calculated for each individual. This information was summarized in Supplementary Table S4.

### Comparisons with other large-scale WGS datasets of Chinese and East Asian populations

The genetic variants in STROMICS were compared with those from Nyuwa (*n* = 2999, http://bigdata.ibp.ac.cn/NyuWa/)^24^, ChinaMAP (*n* = 10,588, http://www.mbiobank.com/)^23^, WBBC (v20211129; 13.9×; the combination of 4535 WGS genomes and 5841 arrays)^26^, gnomAD (v3, *n* = 2604 for East Asians, http://gnomad-sg.org/)^25^. Genetic variants with alternative allele frequency > 0 were applied, and genetic variants that had the same genomic coordinate, reference allele, and alternative allele among different datasets were regarded as identical variants. For each database or genome resource, we excluded the variants with alternative allele frequency = 0.

We also compared the genotype frequency of stroke- and ischemic stroke-susceptible genetic variants among different populations. Although Nyuwa, ChinaMAP, and gnomAD only provided alternative allele count (AC) and total allele number (AN) in the databases, we calculated the alternative and reference allele frequencies and then inferred the frequency for all of the 3 genotypes according to the rule of Hardy-Weinberg Equilibrium.

Among all the 99 genetic variants associated with stroke risk or drug response, 6 variants associated with stroke risk and 2 associated with warfarin response, are absent in at least one of the STROMICS, ChinaMAP, Nyuwa, or WBBC call sets likely due to differences in variant calling criteria (Supplementary Fig. S6 and Table S9).

### Population structure analysis

Autosomal SNVs were applied in PCA and admixture analyses. SNVs with MAF ≥ 1%, call rate ≥ 95%, and *P* value for Hardy–Weinburg Equilibrium > 10^–6^ were extracted. Then, LD was removed by pruning, and *R*^2^ for the remaining SNVs should be < 0.5 in a sliding window of 500 Kb with 1 SNV as a step. After these processes, for PCA on 10,241 patients and Han individuals (*n* = 9947), a total of 887,795 and 887,040 SNVs were applied, respectively (Fig. 3a, b; Supplementary Fig. S9). For PCA on the population that included both STROMICS and subjects in 1KGP3, the autosomal SNVs from STROMICS and subjects in 1KGP3 were merged first, and then the SNVs were filtered by MAF, call rate, and Hardy–Weinburg Equilibrium under identical criteria as above. Then, SNVs in ±500 Kb of the genomic regions that were reported to distort population structure inference were excluded^77^. Finally, a total of 830,137 SNVs were obtained (Supplementary Fig. S13). The top 20 principal components were calculated for each subject using smartpca in EIGENSOFT^78^.

The same datasets of variants were applied in ADMIXTURE analyses with bootstrap = 200^79^. The number of ancestral component K values ranged from 1 to 10 for the population consisting of 10,241 patients in STROMICS. The K values ranged from 1 to 14 for the population that included both STROMICS and subjects in 1KGP3.

In displaying the results of ADMIXTURE analyses, we applied shuffle_popsample_kws function in geneview package of python to prevent the imbalance of sample size among provinces and regions. Thus at most 224 samples in each province, and at most 386 samples in each geographical region of China, were randomly selected when drawing Fig. 3c, d and Supplementary Fig. S13c, d.

### Detection of genetic selection along PC coordinates

Identification of loci under selection through GWAS of eigenvectors was conducted for the 9947 Han individuals in STROMICS. The eigenvectors and PCs were obtained by the aforementioned PCA. Genetic variants in the PC-based selection analysis were SNVs with MAF > 1% and *R*^2^ < 0.9 in a sliding window of 50 SNVs with 5 SNVs as a step among the population of 9947 Han individuals. First, the top 10 eigenvalues and their corresponding eigenvectors were calculated by ProPCA^80^, a component of EigenGWAS^34^. Second, the SNV effects, which were nearly equivalent to F_ST_, were estimated by regressing the genotypes of each SNV with a selected eigenvector. Finally, for each SNV, EigenGWAS provided adjusted *P* values that were corrected by genomic inflation factor λ_GC_^81^ to control population stratification (i.e., drift) and avoid false positive signals for ancestry-informative markers. SNPs with genomic control (GC)-corrected *P* < 5 × 10^–8^ were regarded as loci under selection.

### Genome-wide single variant and gene-based association analysis

We conducted the single-variant and gene-based GWAS analyses for the 18 traits (including 14 quantitative and 4 binary traits, see Supplementary Table S17) among the 10,241 patients using SAIGE (v0.42.1)^82^ and SAIGE-GENE (v1.0.3)^59^, respectively. These quantitative and binary traits were measured as described previously^19^. Considering the statistical power, the variants with MAF > 0.5% were used for single-variant GWAS. Heritability was calculated using Genome-wide Complex Trait Analysis (GCTA, v1.93) based on the same set of variants^83^. Gene-based association analysis was performed using four annotation masks including (1) all the variants with MAF < 0.005; (2) variants with high impact including the LoF, and splice-site variants; (3) variants with high or moderate impact including the LoF, splice-site variants and the inframe-indel/substitution, missense, and stop-loss variants; and (4) variants with high or moderate or low impact including the LoF, splice-site variants, the inframe-indel/substitution, missense variants, stop-loss variants, the synonymous, UTR, and stop retained variants in each gene (Supplementary Table S3). For (2)–(4), three rounds of gene-based association analyses were conducted by applying variants with MAF < 1%, 0.1%, and 0.01%. Aggregated *P*-value of the nine combinations of tests (three annotation masks and three MAF settings) were computed using the Cauchy combination method implemented in SAIGE-GENE.

For the 14 quantitative traits (10 lipid traits, VB9, HCY, HCY, and eGFR), the values were first transformed to a standard normal distribution by quantile transformation. Age, gender, history of stroke, days between disease onset and blood sampling of the patient, and the first five principal components from PCA analysis were included as covariates in both single-variant and gene-based analysis. For the 4 qualitative traits (DRINK, Smoking, AIS-DWI, and sEICAS), age, gender, history of stroke, and the first five principal components from PCA were included as covariates. DRINK was defined as the drinkers who drank ≥ 2 standard alcohol consumption/per day. Smoking was defined as current smokers. AIS-DWI was defined as acute ischemic infarction which was identified by DWI. sEICAS was defined as TIA or ischemic stroke attributed to 50%–99% atherosclerotic stenosis of an extracranial artery or a major intracranial artery.

For replication of single-variant association discoveries, we searched the lead SNP and its proxies (*R*^2^ > 0.8 in 1KGP3 East Asian population) across three databases (GWAS catalog^51^, OpenGWAS^54^, Phenoscanner^52,53^). We identified the studies with the greatest sample size among the East Asian population and downloaded the summary statistics. We reported the summary statistics for the lead SNP or its proxy in Supplementary Table S18. If no GWAS was conducted among the East Asian population, we reported the summary statistics for the studies with the largest sample size among the other populations. We called a lead SNP or its proxy as replicated if it has the same effect direction between the STROMICS analysis and the replication dataset and a *P*-value < 0.05 divided by the number of loci with the replication dataset (*n* = 29), namely, *P* < 1.72 × 10^–3^.

### Co-localization analysis to detect pleiotropic loci

To understand whether the significant genetic loci may have a pleiotropic effect on other biochemical traits assayed in the study, we tested the probability that the genetic determinants of a specific trait were shared with the rest of the other traits using co-localization analyses, as implemented in coloc^84^. We performed the analysis for each of the genome-wide significant loci (a 1 Mbp window centering on the lead SNP, *n* = 46, *P* < 5 × 10^–8^) separately. For each locus, if any two traits with a posterior probability (PP) of a shared single causal signal > 0.8 (H4 > 0.8), we further identified the SNP that was most likely causal for the shared signal (SNP.PP.H4 > 0.8). In total, six loci were identified to have exhibited a pleiotropic effect. The likely causal SNP and the H4 probability were presented in Supplementary Table S19. For each SNP, the GWAS *P*-values of the 14 biochemical traits were presented in Supplementary Fig. S19.

### Identification of known P/LP variants in the WGS dataset of STROMICS

To identify the P/LP genetic variants, we retrieved the ClinVar dataset (release 20211218 at https://ftp.ncbi.nlm.nih.gov/pub/clinvar/vcf_GRCh38/weekly/). A genetic variant in the ClinVar database would be regarded as a known P/LP if it fulfilled these criteria:

1. the variant must be recorded by MedGen;
2. the annotations of the variant in ClinVar should not contain any one of these strings including “conflicting”, “benign”, “likely benign”, or “uncertain significance”.

A total of 149,355 known P/LP variants were obtained from ClinVar database. Then we identified the genetic variants in STROMICS dataset that had the same genomic coordinate, reference allele, and alternative allele as the P/LP variants in ClinVar. The alternative allele counts of these reported P/LP variants were also calculated in the STROMICS (Supplementary Table S22).

### Annotation of genetic variants in *NOTCH3* gene

A total of 2265 genetic variants were mapped to *NOTCH3* gene in STROMICS (Supplementary Table S24). ClinVar database recorded 188 of the 2265 genetic variants, including 187 SNVs (9 were P/LP variants) and 1 indel. All of these 188 genetic variants had been recorded in dbSNP.

The information on domains of *NOTCH3* was retrieved from UniProt (https://www.uniprot.org/uniprot/Q9UM47) and SMART database^85^.

Then, LoF, splicing variants, as well as missense and non-frameshift variants that resulted in substitution, loss, or gain of Cys in the NOTCH3 protein, were denoted as CADASIL-susceptible variants. All of the 87 carriers for these variants were extracted, and they carried a total of 49 CADASIL-susceptible variants (Supplementary Tables S23–S25). Each of the 87 carriers had only 1 CADASIL-susceptible variant in the genome. One LoF variant was a multi-nucleotide polymorphism (MNP) that was integrated from three indels (patient ID 80 in Supplementary Table S25).

To estimate the credibility of CADASIL-susceptible variants in *NOTCH3*, 73 out of the 87 carriers underwent target-capture NGS (mentioned above). All of the CADASIL-susceptible variants in 73 carriers were validated (Supplementary Table S25).

### Brain imaging

Brain imaging was conducted for patients, including brain MRI (T1 weighted, T2 weighted, Fluid-attenuated Inversion Recovery (FLAIR), Turbo Inversion Recovery Magnitude (TIRM), DWI with Apparent Diffusion Coefficient (ADC) maps, Magnetic Resonance Angiography (MRA), T2*/Susceptibility Weighted Imaging (SWI)) or CT. If the patients were contraindicated to MRI, they only accepted the CT scan^19^. Among the 87 carriers for CADASIL-susceptible variants in the study, the brain MRI of 6 patients was not available. A radiologist and a neurologist who were blind to the genetic results of *NOTCH3*, analyzed all the available scans and assessed the following features:

1. Presence and severity of DWMH and PVH on FLAIR or T2-weighted images were evaluated according to Fazekas scale^86^. The grades of DWMH were grouped as being grade 0 (absence), grade 1 (punctate foci), grade 2 (beginning confluence), or grade 3 (large confluent areas). The grades of PVH were grouped as being grade 0 (absence), grade 1 (caps or pencil-thin lining), grade 2 (smooth halo), or grade 3 (irregular PVH extending into deep white matter). The severity of WMH load was the sum of DWMH and PVH, and was categorized as mild (summed score: 0–2), moderate (summed score: 3–5), or severe (summed score: 6).
2. Presence of hyperintense lesions (FLAIR and T2-weighted images) in the anterior temporal lobe white matter.
3. Presence of hyperintense lesions (FLAIR and T2-weighted images) in the external capsules white matter.
4. Presence of lacunar lesions is defined as focal hyperintensities on T2-weighted images, with the corresponding hypointensity on T1-weighted images, and larger than 3 mm in size^87^.
5. Presence of brain microbleeds defined as small, rounded, or circular, hypointense lesions within brain parenchyma with margins ranging from 2 mm to 10 mm in size on GRE T2* or weighted SWI images^88^.
6. Presence of brain atrophy is defined as global atrophy (sulcal and ventricular dilation) or medial temporal lobe atrophy^89,90^.

## Supplementary information

Supplementary information

Supplementary Tables

## Data Availability

The summary information from the STROMICS, including the position, reference allele, alternative allele, allele frequencies, and the genetic association with the 18 investigated traits of the 135.59 million variants can be accessed through the STROMICS browser (http://www.stromics.org.cn). Researchers can gain access to the data online. The raw sequencing data from the STROMICS have been deposited in the Genome Sequence Archive for Human (https://ngdc.cncb.ac.cn/gsa/) at the National Genomics Data Center, Beijing Institute of Genomics, Chinese Academy of Sciences, under the accession number (HRA001351) and the database of the China National Clinical Research Center for Neurological Diseases in Beijing Tiantan Hospital, following the regulations of the Human Genetic Resources Administration of China (HGRAC) (2022BAT1860 and 2023BAT0441). In compliance with the regulations of the Ministry of Science and Technology of the People’s Republic of China, the raw sequencing data contain information unique to an individual and thus require controlled access. Further analysis of sequencing data will be made available for collaborating researchers upon request, in compliance with the HGRAC’s approval.
