# Supplementary material for: The STROMICS genome study: deep whole-genome sequencing and analysis of 10K Chinese patients with ischemic stroke reveal complex genetic and phenotypic interplay
Source: Cell Discov. 2023 Jul 21;9:75. doi: 10.1038/s41421-023-00582-8 (PMC10362040; doi:10.1038/s41421-023-00582-8)
Supplement: Supplementary file 1 — Supplementary information [file 41421_2023_582_MOESM1_ESM.pdf]

Supplementary information for

The STROMICS Genome Study: Deep whole-genome sequencing and analysis of 10K Chinese patients with ischaemic stroke

Si Cheng[1,2,3,4,5], Zhe Xu[1,2,5], Shengzhe Bian[6], Xi Chen[7], Yanfeng Shi[1,2,5], Yanran Li[1,2,5], Yunyun Duan[8], Yang Liu[1,2,5], Jinxi Lin[1,2], Yong Jiang[1,2], Jing Jing[1,2,9], Zixiao Li[1,2], Yilong Wang[1], Xia Meng[1,2], Yaou Liu[8], Mingyan Fang[10], Xin Jin[10], Xun Xu[10,11], Jian Wang[10,12], Chaolong Wang[13], Hao Li[1,2,5], Siyang Liu[6,10], Yongjun Wang[1,2,3,4,5]

[1] Department of Neurology, Beijing Tiantan Hospital, Capital Medical University, Beijing 100070, China;
[2] China National Clinical Research Center for Neurological Diseases, Beijing 100070, China;
[3] Changping Laboratory, Beijing 100000, China;
[4] Clinical Center for Precision Medicine in Stroke, Capital Medical University, Beijing 10069, China;
[5] Center of excellence for Omics Research (CORe), Beijing Tiantan Hospital, Capital Medical University, Beijing 10070, China;
[6] School of Public Health (Shenzhen), Sun Yat-sen University, Shenzhen 518107, Guangdong, China;
[7] BGI-Tianjin, BGI-Shenzhen, Tianjin 300308, China;
[8] Department of Radiology, Beijing Tiantan Hospital, Capital Medical University, Beijing 10070, China;
[9] Tiantan Neuroimaging Center of Excellence, Beijing 10070, China;
[10] BGI-Shenzhen, Shenzhen 518083, Guangdong, China;
[11] Guangdong Provincial Key Laboratory of Genome Read and Write, BGI-Shenzhen, Shenzhen 518120, Guangdong, China;
[12] James D. Watson Institute of Genome Sciences, Hangzhou 310058, China;
[13] Department of Epidemiology and Biostatistics, Ministry of Education Key Laboratory of Environment and Health, State Key Laboratory of Environmental Health (Incubating), School of Public Health, Tongji Medical College, Huazhong University of Science and Technology, Wuhan 430030, China

These authors contributed equally as co-first authors: Si Cheng, Zhe Xu

Those authors contributed equally as co-second authors: Shengzhe Bian, Xi Chen, Yanfeng Shi

Correspondence:
Siyang Liu (liusy99@mail.sysu.edu.cn)
Yongjun Wang (yongjunwang@ncrcnd.org.cn)

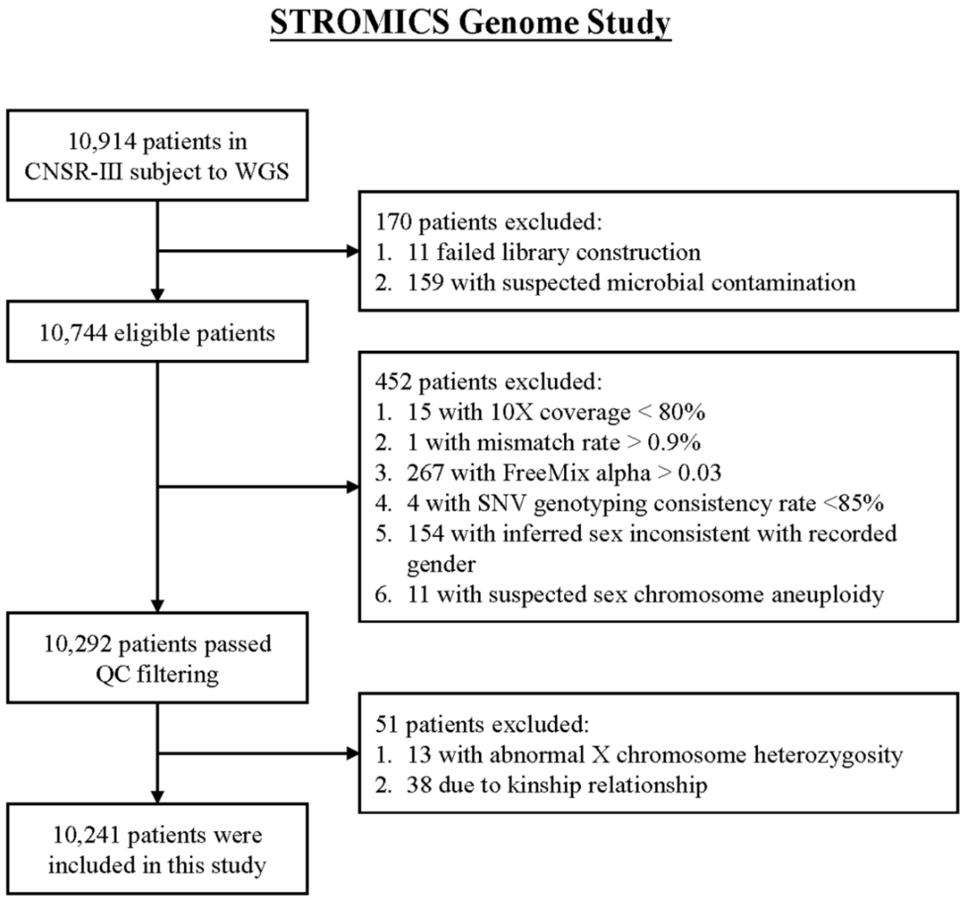

Fig. S1 Flow chart and quality control of 10,241 samples in STROMICS Genome Study. CNSR-Ⅲ: the Third China National Stroke Registry. SNV: single nucleotide variation. QC: quality control.

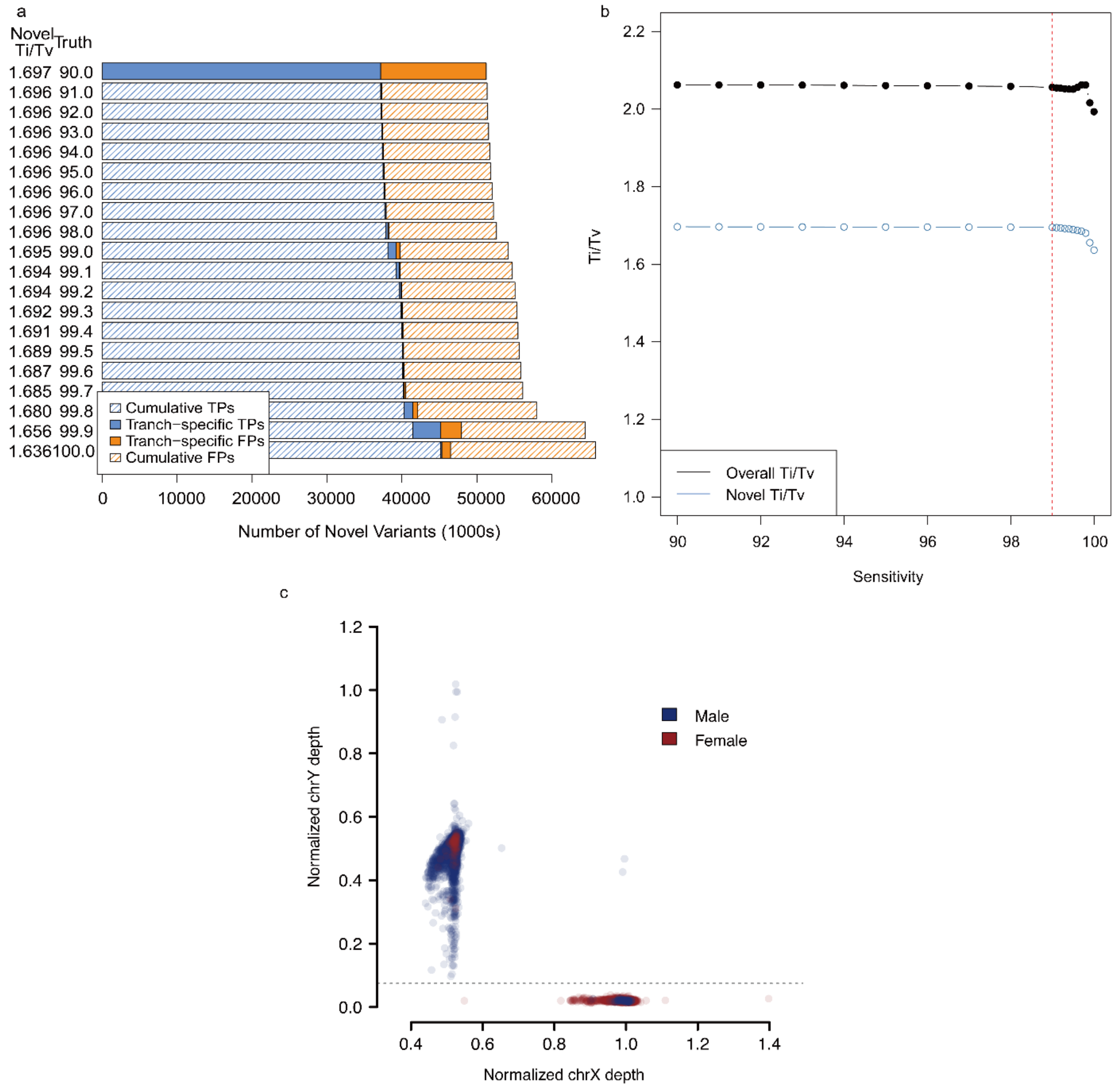

Fig. S2 VQSR and sex check for WGS data.

a Tranche sensitivity plot of novel SNVs as reported by the VQSR model fitting. Truth, truth sensitivity. Ti/Tv, transition/transversion ratio.

b Truth sensitivity cutoff for VQSR in STROMICS. The red dashed line denotes a truth sensitivity of 99.0. This cutoff was chosen because the curve of both overall and novel variants Ti/Tv began to decline after truth sensitivity reached 99.0. Novel variants in this figure were defined according to whether the SNV existed in the GATK bundle resource. The Ti/Tv value in this figure was calculated during VQSR. Notably, the Ti/Tv values in the results of the manuscript were produced after all of the quality control procedures including microbial contamination evaluation, abnormal coverage and mismatch rate filter, sex check, VQSR, kinship relatedness filter, and genotype filter.

c Sex check for WGS data. The normalized sequencing depth of chromosomes X and Y was applied as the coordinate. The reported gender in the electronic data capture (EDC) system was shown. The male and female patients formed distinct clusters in the plot. The dashed line

indicated normalized chromosome Y depth 0.075, which is regarded as the threshold to separate male and female patients. Thus 154 patients with inconsistency between inferred sex and recorded gender, 13 patients of abnormal X chromosome heterozygosity, and 11 patients with suspected sex chromosome aneuploidy were resolved.

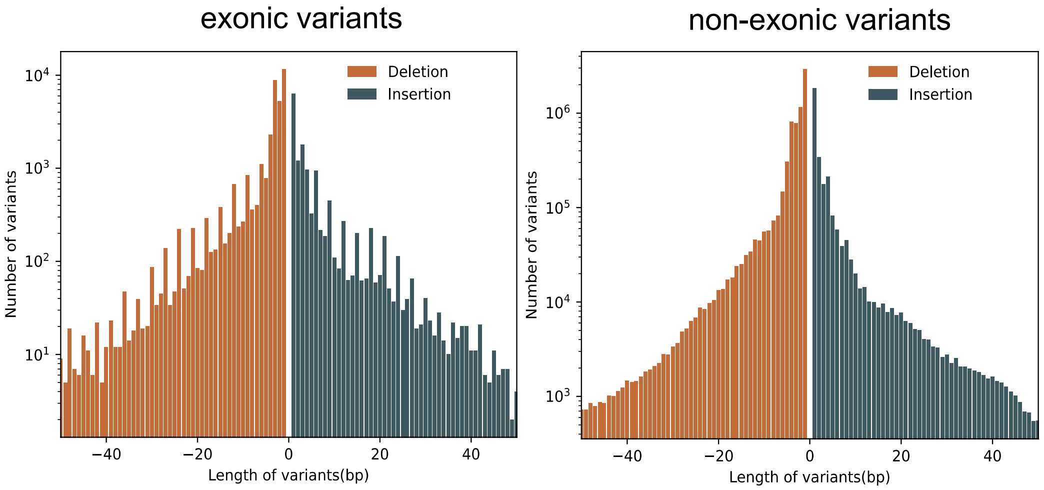

Fig. S3 Length and number distribution of STROMICS exonic and non-exonic indels.

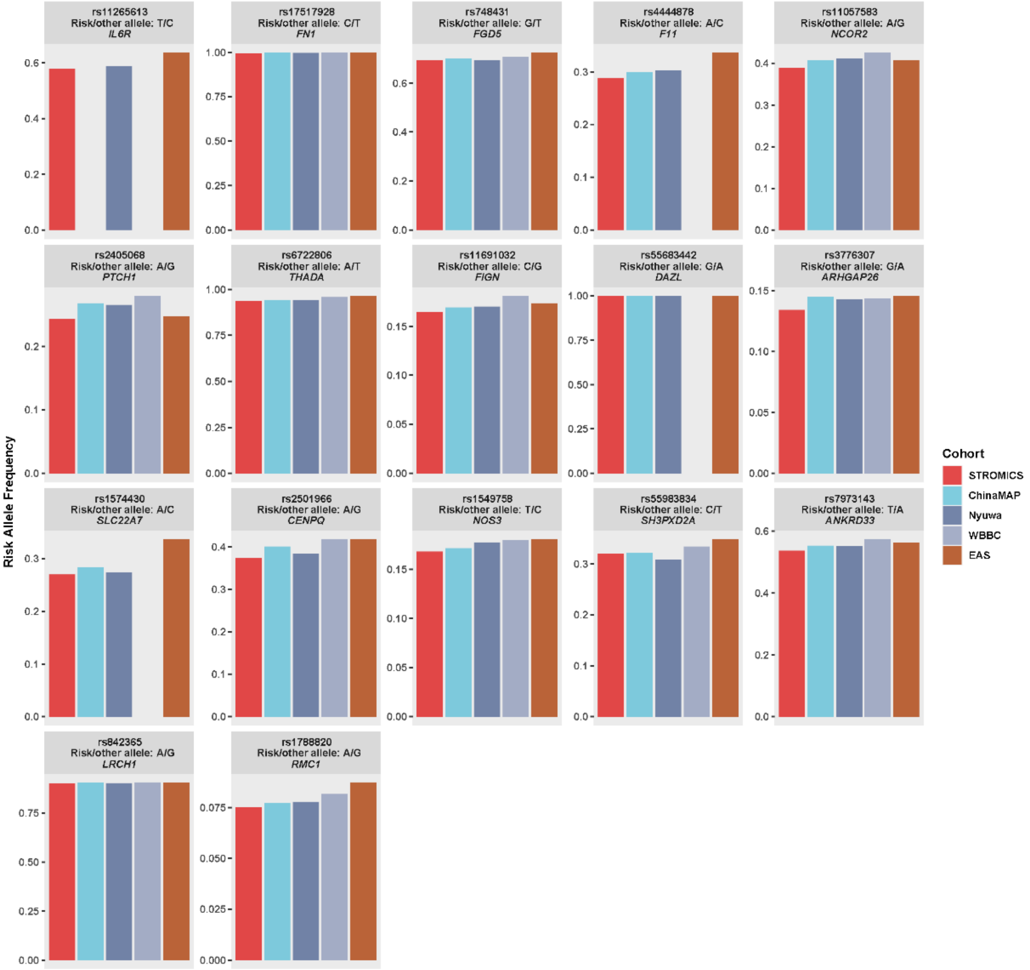

Fig. S4 Risk allele frequency of 17 genetic variants associated with stroke risk occurrence but demonstrated unexpectedly lower allele frequency in STROMICS compared to other Chinese reference data sets.

The control datasets include China Metabolic Analytics Project (ChinaMAP), NyuWa Genome resource (Nyuwa), Westlake BioBank for Chinese (WBBC) pilot project, and East Asians (EAS) from The Genome Aggregation Database (gnomAD). Nearby genes, risk, and reference alleles were demonstrated. This figure was related to Supplementary Table S9.

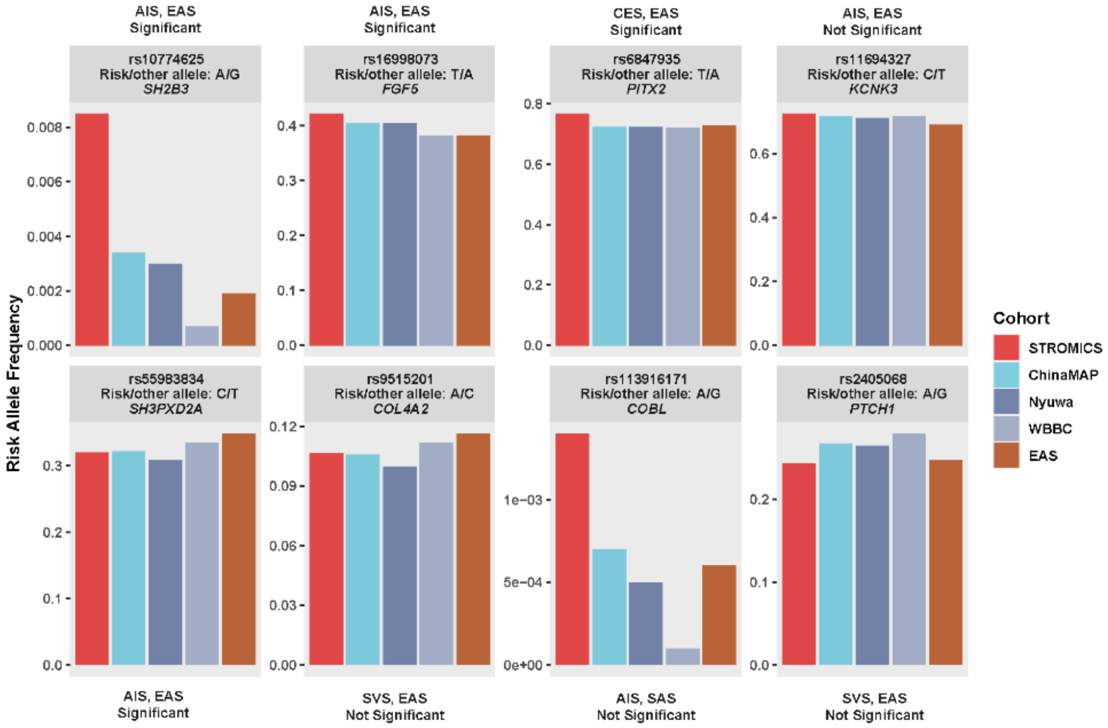

Fig. S5 Comparison of risk allele frequency of 8 genetic variants known to be associated with stroke occurrence in non-European populations among STROMICS, China Metabolic Analytics Project (ChinaMAP), NyuWa Genome resource (Nyuwa), Westlake BioBank for Chinese (WBBC) pilot project, and East Asians (EAS) from The Genome Aggregation Database (gnomAD).

Nearby genes, risk, other alleles, and associated stroke types were demonstrated. Statistic details were summarized in Supplementary Table S9. AS, any stroke. AIS, any ischaemic stroke. LAS, large artery stroke. CES, cardioembolic stroke. SVS, small-vessel occlusion.

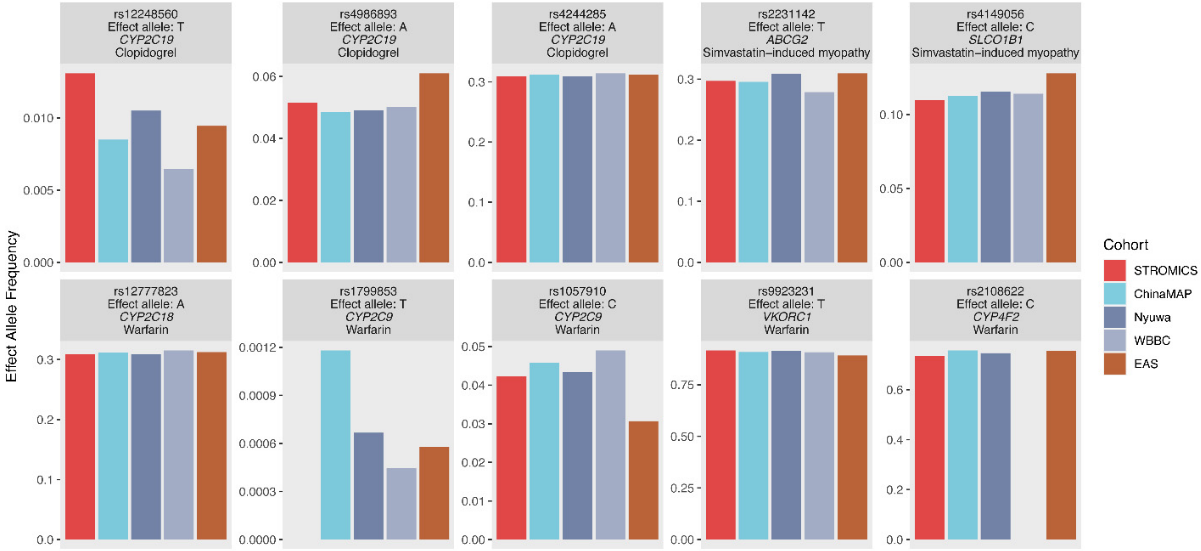

Fig. S6 Effect allele frequencies of the 10 genetic variants associated with drug metabolism among STROMICS, ChinaMAP, NyuWa, WBBC, and EAS from gnomAD.
For each variant, the effect allele, the drug, and the associated genes were shown.

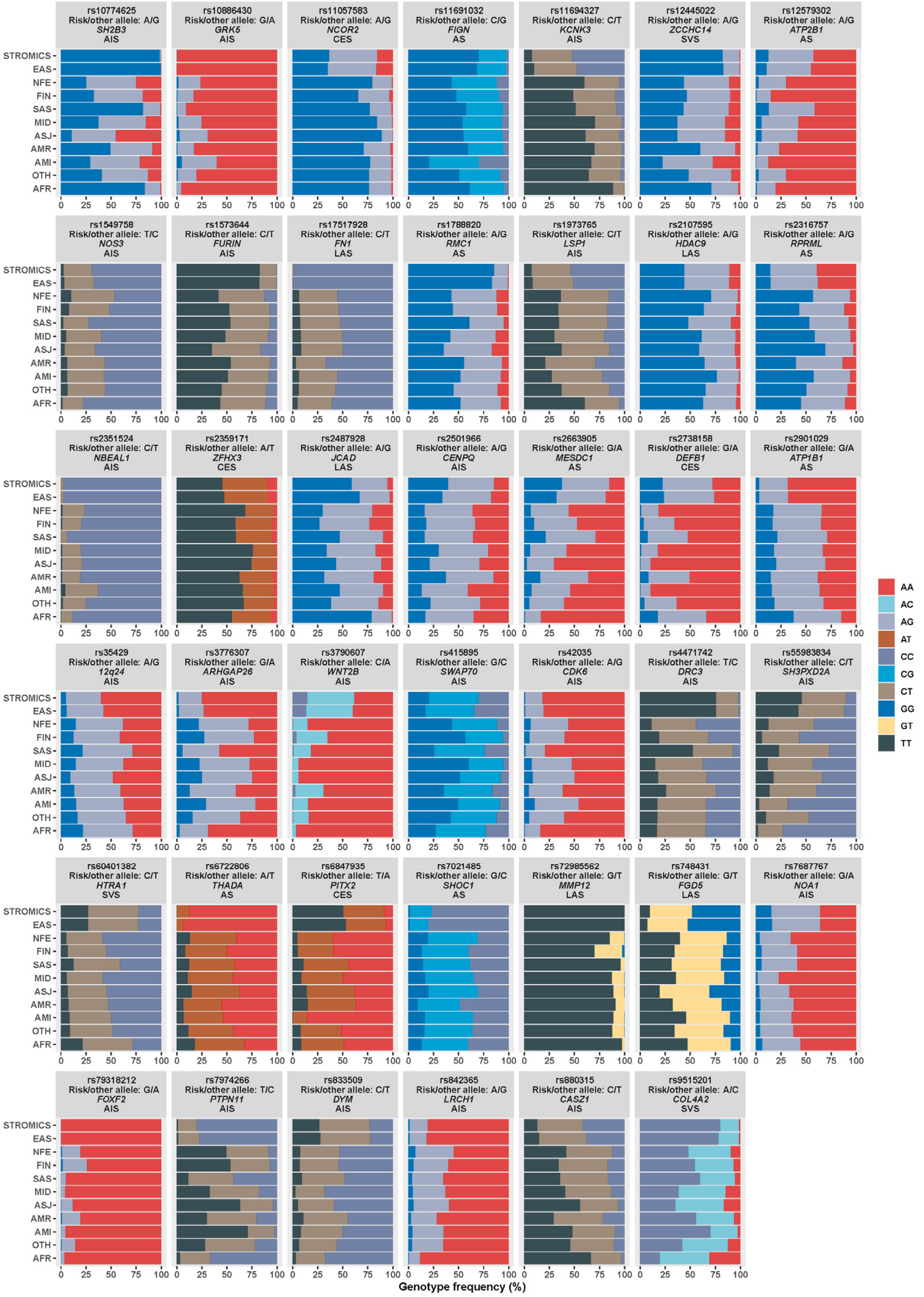

Fig. S7 Genotype frequencies of 41 stroke risk variants that demonstrated the most significant ancestral difference in allele frequency between STROMICS and the non-Finnish European populations, related to Supplementary Table S11.

Shown are the genotype frequencies obtained from the STROMICS and the Genome Aggregation Database (gnomAD). The nearest genes or the genomic regions, associated stroke type or subtype, and the risk/reference alleles were listed for each variant.
EAS: East Asians. NFE: Non-Finnish Europeans. FIN: Finnish. SAS: South Asians. MID: Middle Eastern population. ASJ: Ashkenazi Jewish. AMR: Latino. AMI: Amish. OTH: population of other ancestry. AFR: Africans/African-Americans. AS: any stroke. AIS: any ischaemic stroke. LAS: large-artery atherosclerotic stroke. CES: cardioembolic stroke.

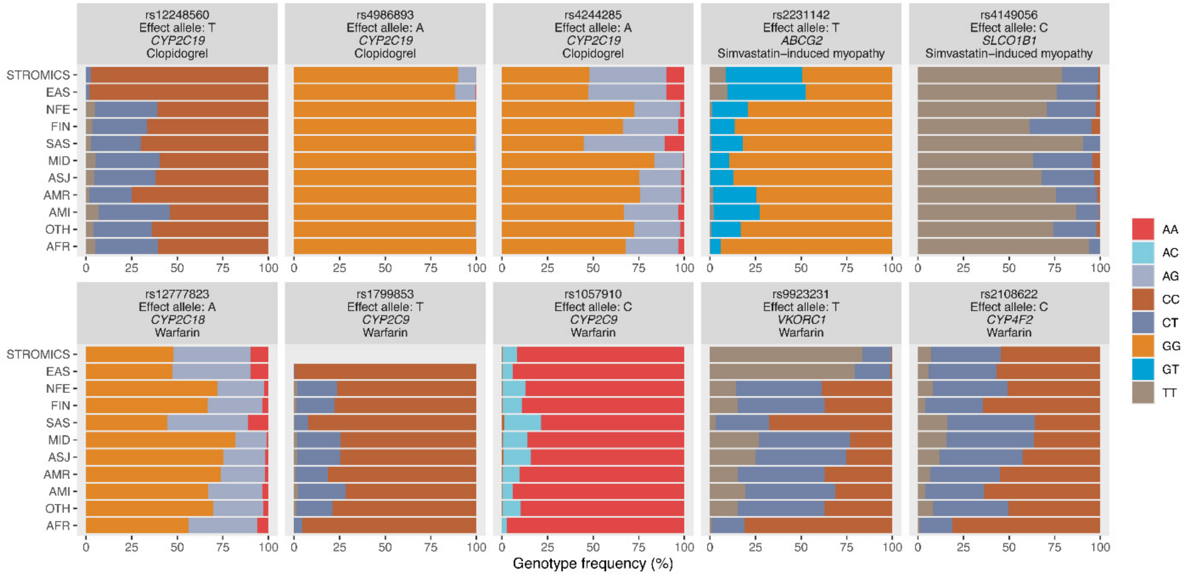

Fig. S8 Genotype frequencies of the 10 genetic variants associated with drug metabolism among STROMICS and other populations in gnomAD.

For each variant, the effect allele, the drug, and the associated genes were shown.
EAS: East Asians. NFE: Non-Finnish Europeans. FIN: Finnish. SAS: South Asians. MID: Middle Eastern population. ASJ: Ashkenazi Jewish. AMR: Latino. AMI: Amish. OTH: population of other ancestry. AFR: Africans/African-Americans.

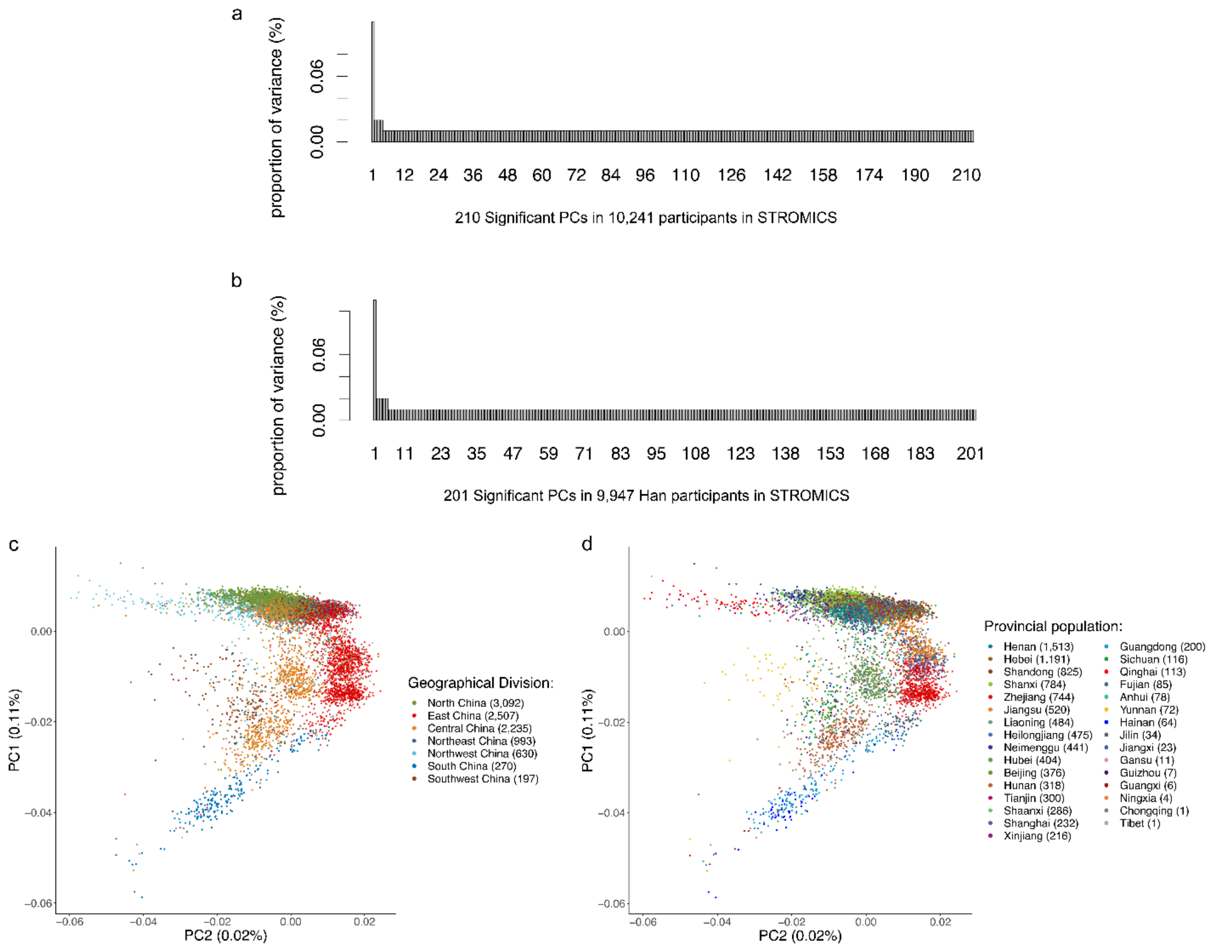

Fig. S9 The proportion of variance distribution of significant principal components and PCA of Han individuals (N = 9,947) in STROMICS.

a-b Barplot of the proportion of variance explained for the 210 and 201 significant principal components in 10,241 patients (**a**) and 9,947 Han individuals (**b**) in STROMICS (*P*-value of Tracy-Widom test < 0.05). **c-d** Scatter plots drawn among different PCs. Each point represents one Han individual and is placed according to the eigenvectors. Individuals from different geographical regions (**c**) or different provinces (**d**) were represented by different colors. For **c** and **d**, the PCA was conducted using 887,040 autosomal SNVs (Materials and methods). The proportion of variance explained by each PC was listed after the PCs.

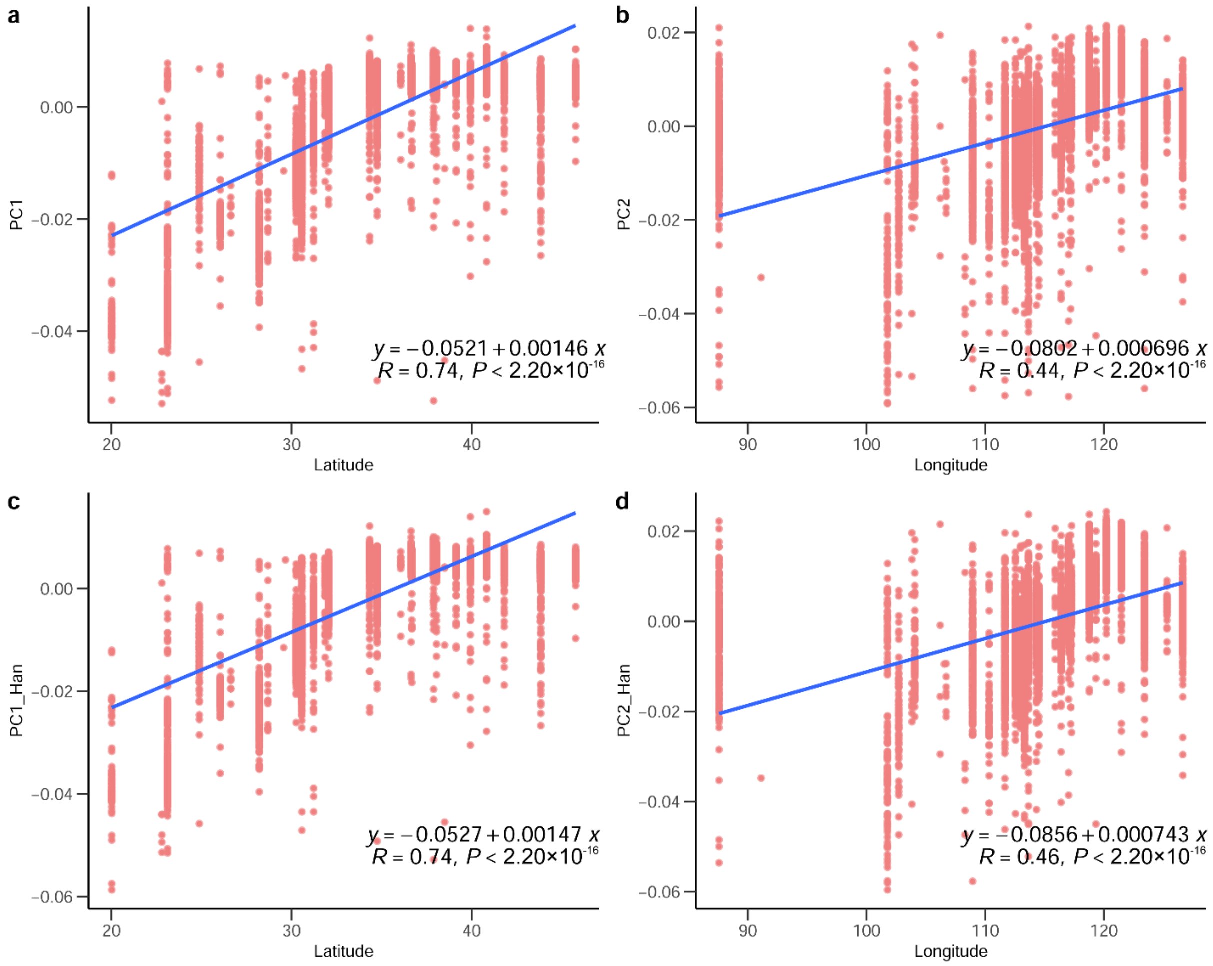

Fig. S10 Pearson correlation between principal components (PCs) with latitude and longitude.

a correlation between PC1 and latitude in 10,241 participants in STROMICS.
b correlation between PC2 and longitude in 10,241 participants in STROMICS.
c correlation between PC1 and latitude in 9,947 Han individuals in STROMICS.
d correlation between PC2 and longitude in 9,947 Han individuals in STROMICS.
We calculated the correlation coefficient between PC1/PC2 and latitude/longitude coordinates of the capital of every province, which were obtained from Baidu Map API (http://api.map.baidu.com/lbsapi/getpoint/index.html), a BD09 coordinate system.

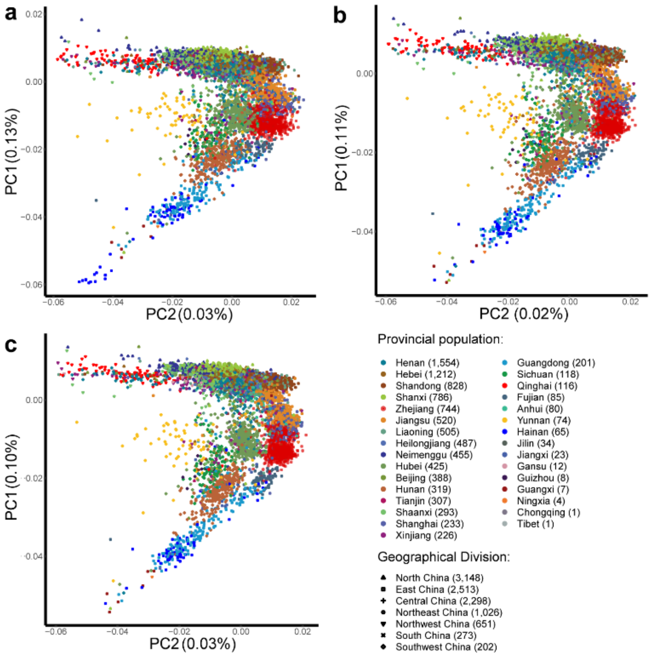

Fig. S11 PCA of STROMICS participants (N = 10,241) at different MAF cutoffs.
a PCA that was conducted using biallelic SNVs with MAF above 5% (394,437 SNVs).
b PCA that was conducted using biallelic SNVs with MAF above 1% (887,795 SNVs).
c PCA that was conducted using biallelic SNVs with MAF above 0.5% (1,283,693 SNVs).
Each point represents one participant and is placed according to their eigenvectors. Sample size in each province for PCA using SNVs with MAF > 0.5% was shown.

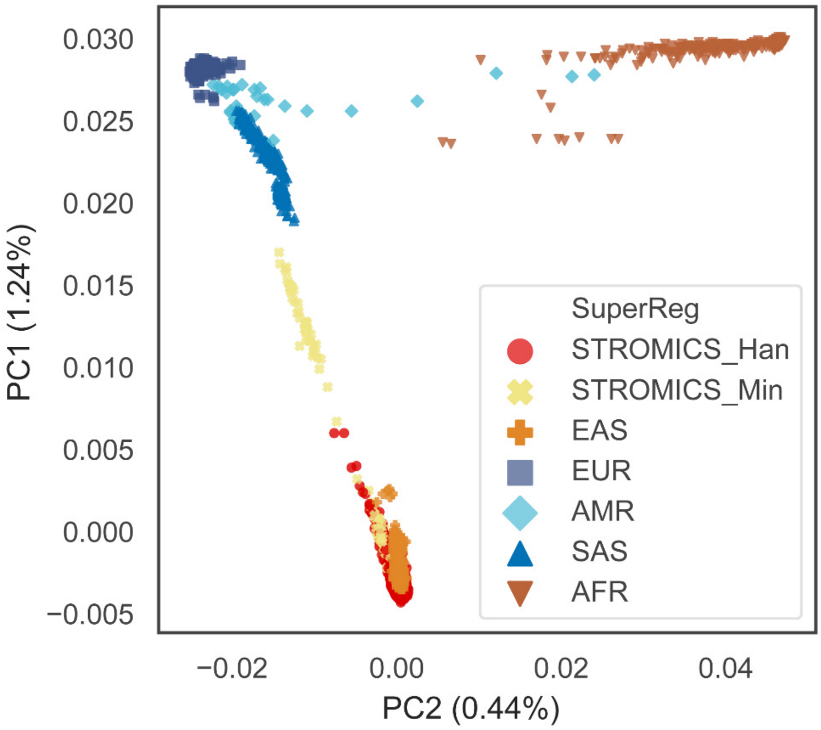

Fig. S12 PCA of STROMICS participants (N = 10,241) and the 1000 Genome Project Phase 3 individuals (N = 2,503).
A total of 830,137 SNVs were applied (see Materials and methods for detailed procedures).

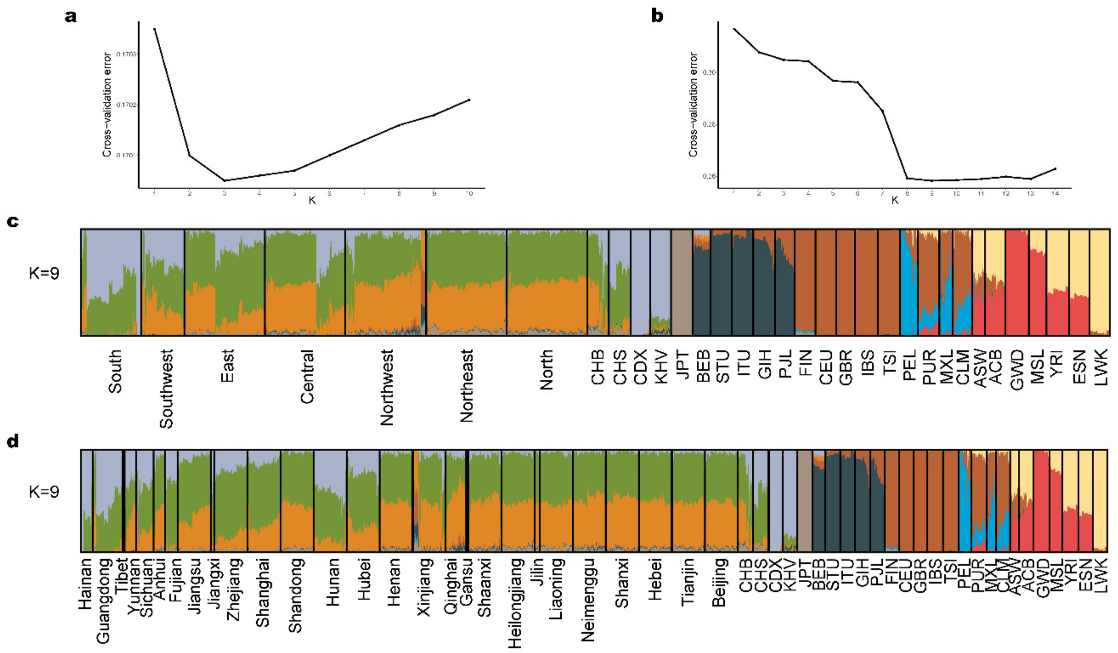

Fig. S13 ADMIXTURE analysis of the STROMICS participants (N = 10,241) and the 1000 Genome Project Phase 3 individuals (N = 2,503).

a Line chart of cross validation (CV) error for the STROMICS participants (N = 10,241) with the smallest CV error occurring at K = 3.

b Line chart of CV error for the STROMICS participants (N = 10,241) and the 1000 Genome Project Phase 3 individuals (N = 2,503) with the smallest CV error occurring at K = 9.

c Distribution of the 9 ancestry components in STROMICS participants (N = 10,241) of different geographical regions and the 1000 Genome Project Phase 3 individuals (N = 2,503) as inferred using the ADMIXTURE for K = 9.

d Distribution of the 9 ancestry components in STROMICS participants (N = 10,241) of different provinces and the 1000 Genome Project Phase 3 individuals (N = 2,503) as inferred using the ADMIXTURE for K = 9.

In **c** and **d**, the 1000 Genome Project Phase 3 individuals (N = 2,503) are organized by population.

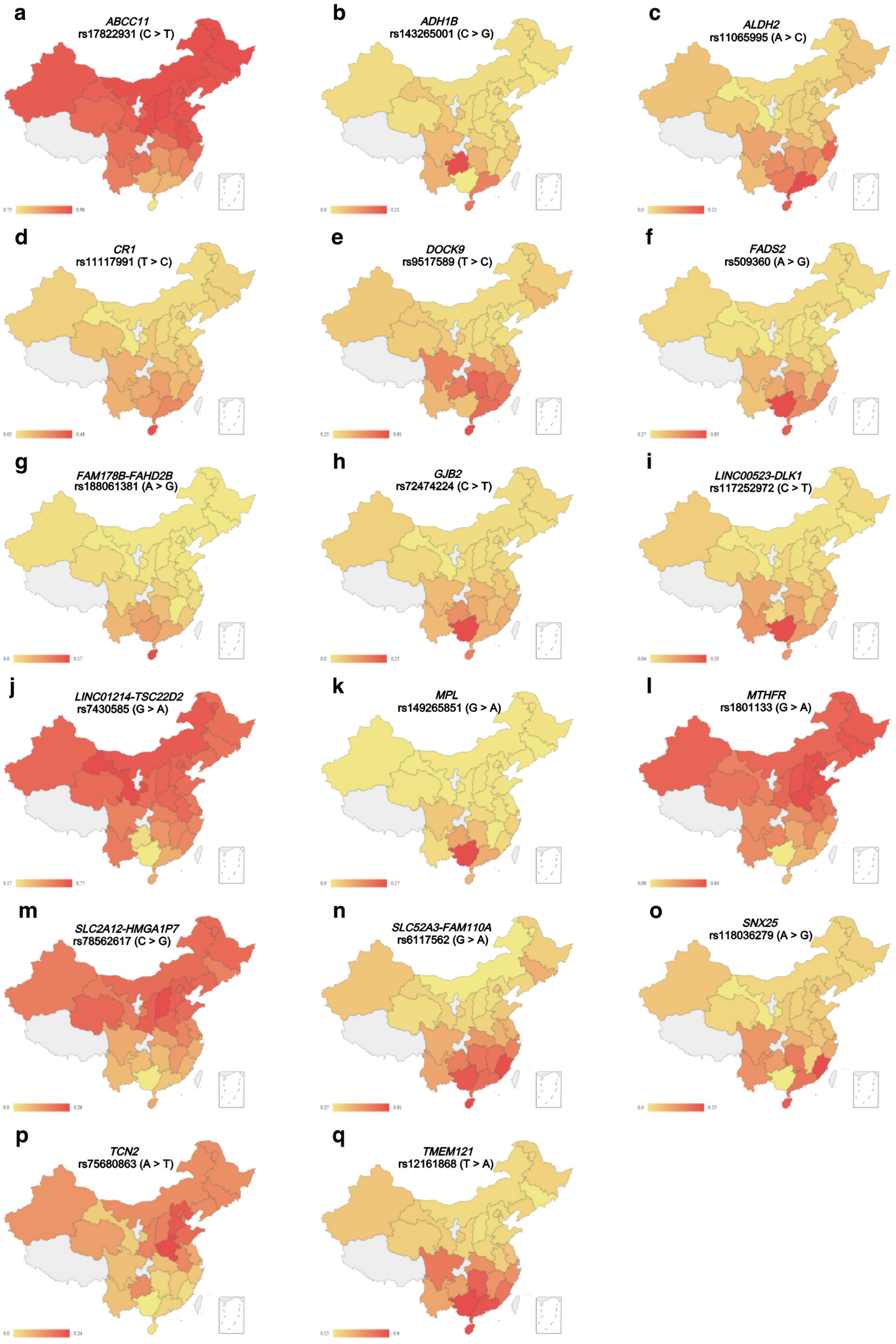
a
ABCC11
rs17822931 (C > T)
b
ADH1B
rs143265001 (C > G)
c
ALDH2
rs11065995 (A > C)
d
CR1
rs11117991 (T > C)
e
DOCK9
rs9517589 (T > C)
f
FADS2
rs509360 (A > G)
g
FAM178B-FAHD2B
rs188061381 (A > G)
h
GJB2
rs72474224 (C > T)
i
LINC00523-DLK1
rs117252972 (C > T)
j
LINC01214-TSC22D2
rs7430585 (G > A)
k
MPL
rs149265851 (G > A)
l
MTHFR
rs1801133 (G > A)
m
SLC2A12-HMGA1P7
rs78562617 (C > G)
n
SLC52A3-FAM110A
rs6117562 (G > A)
o
SNX25
rs118036279 (A > G)
p
TCN2
rs75680863 (A > T)
q
TMEM121
rs12161868 (T > A)

Fig. S14 Alternative allele frequency distribution of the 17 SNVs that demonstrated genome-wide significance across PC1, related to Fig. 3e.
(**a**-**q**) The identifier of SNVs and corresponding genes were shown. The provinces were colored according to the alternative allele frequency of the SNVs.

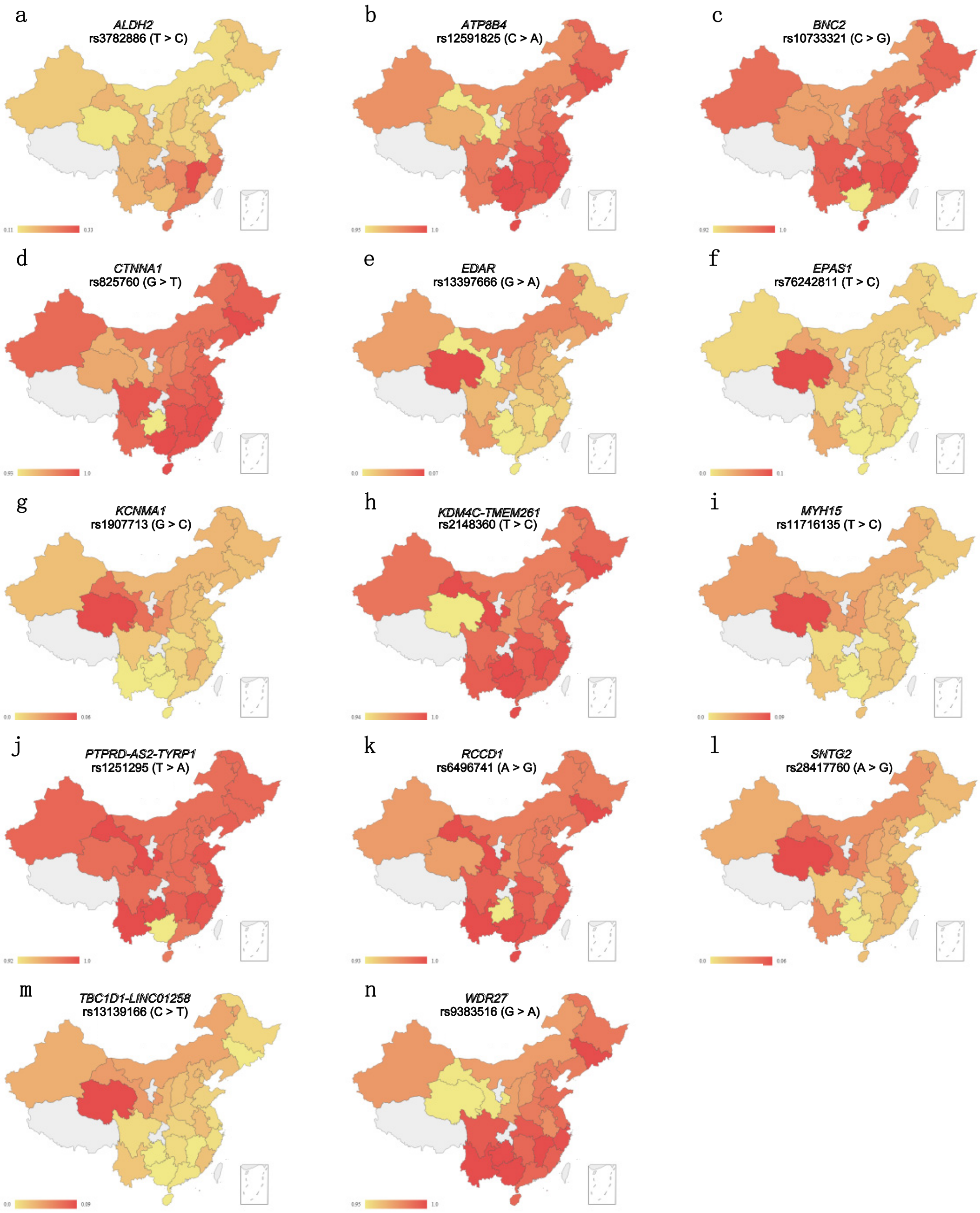

Fig. S15 Alternative allele frequency distribution of the 14 SNVs that demonstrated genome-wide significance across PC2, related to Fig. 3e.
(**a**-**n**) The identifier of SNVs and corresponding genes were shown. The provinces were colored according to the alternative allele frequency of the SNVs.

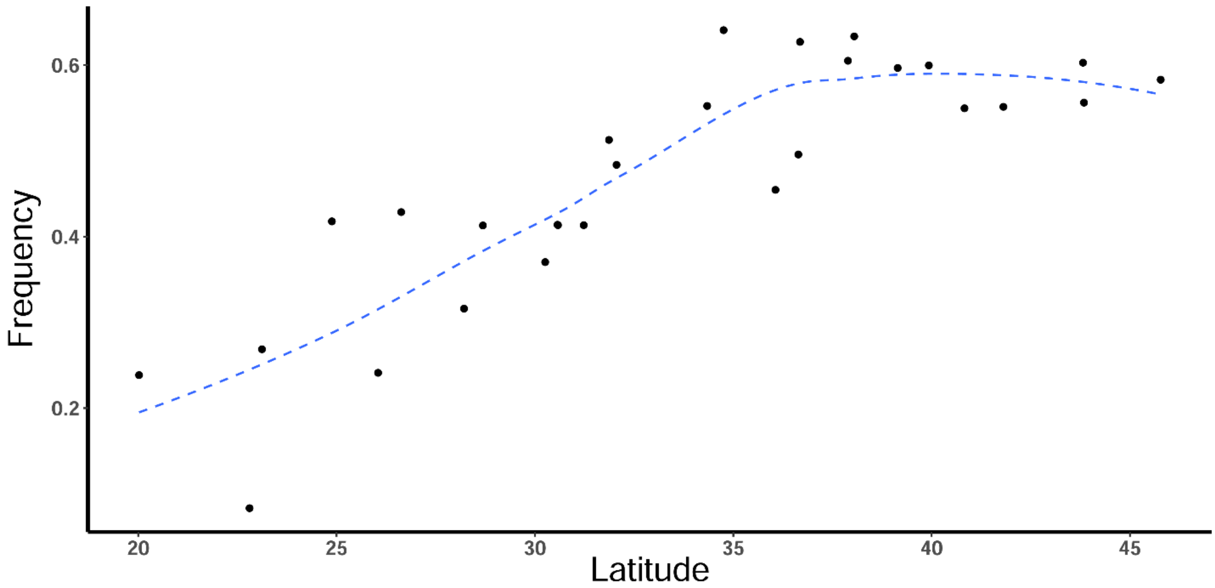

Fig. S16 A allele frequency of rs1801133 as a function of latitude, related to Fig. 3f. Latitude coordinates of the capital of every province, which were obtained from Baidu Map API (http://api.map.baidu.com/lbsapi/getpoint/index.html), were applied in this figure.

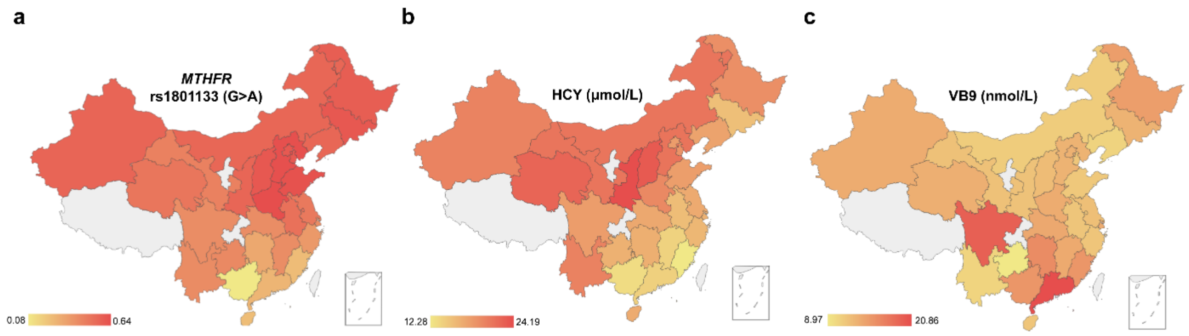

Fig. S17 A allele Frequency of rs1801133 (*MTHFR*), level of homocysteine (HCY), and Vitamin B9 (VB9), related to Fig. 3g, h.
a Geographical distribution of the A allele frequency of the SNV rs1801133 (chr1: 11796321) in the *MTHFR* and *CLCN6* locus under genetic selection in STROMICS population.
b Level of HCY (umol/L) in serums of 10,241 patients in STROMICS.
c Level of VB9 (nmol/L) in serums of 10,241 patients in STROMICS.
Provinces whose sample size was less than 5 were filled with gray color. The provinces were colored according to A allele frequency, level of HCY, and VB9 in serums (color scale in the lower left corner for each figure).

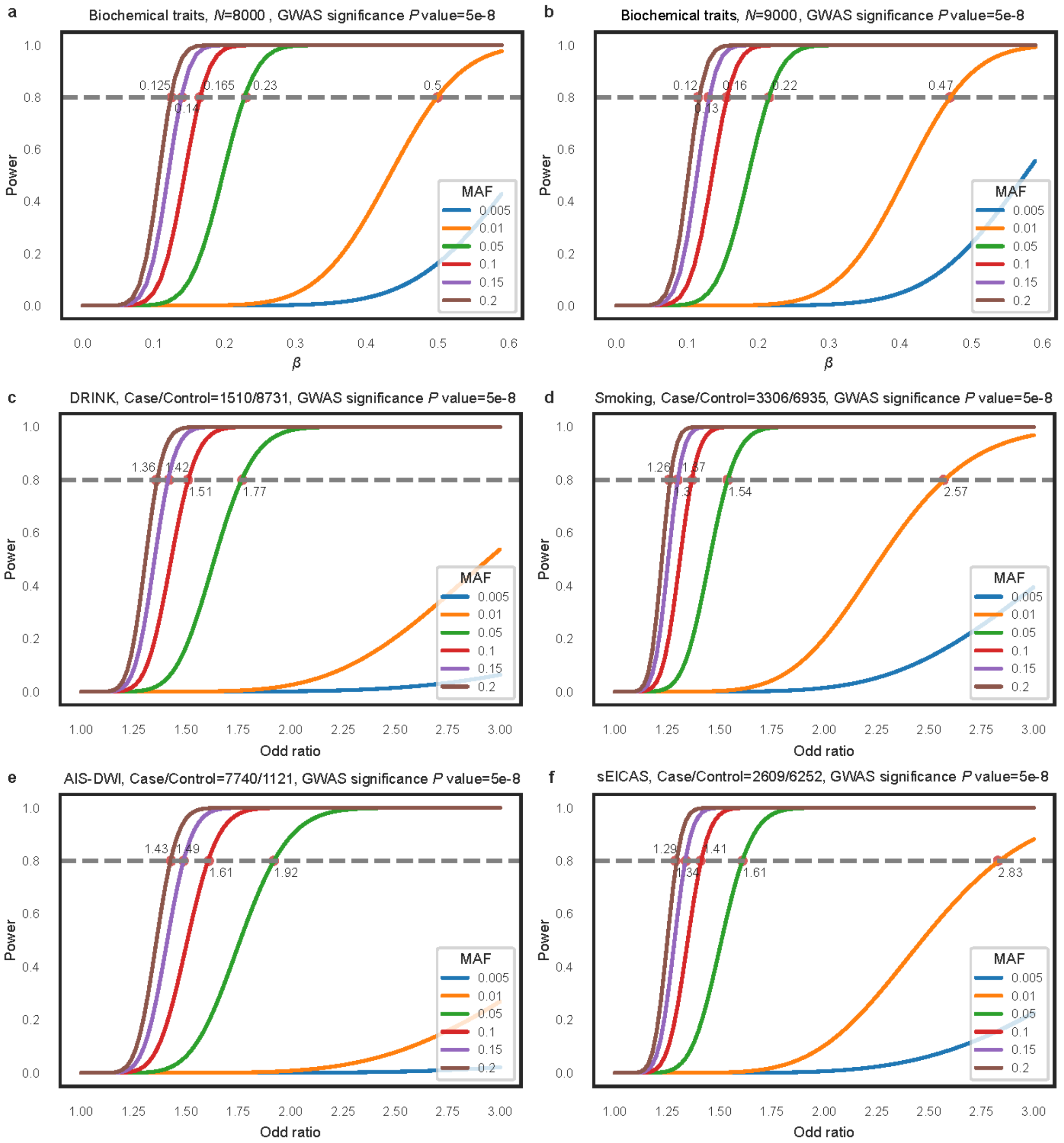

Fig. S18. Power analysis for genome-wide association test.
For quantitative traits, the power analysis was conducted using formula (3) in Visscher et al., AJHG, 2017 (PMID: 28686856), assuming a significance level of 5×10^{-8}. For qualitative traits, the power analysis was conducted using the same formula as the GAS Power Calculator which was published in Box 5 in Sham et al., NGR, 2014 (PMID: 24739678), also assuming a significance level of 5×10^{-8}.

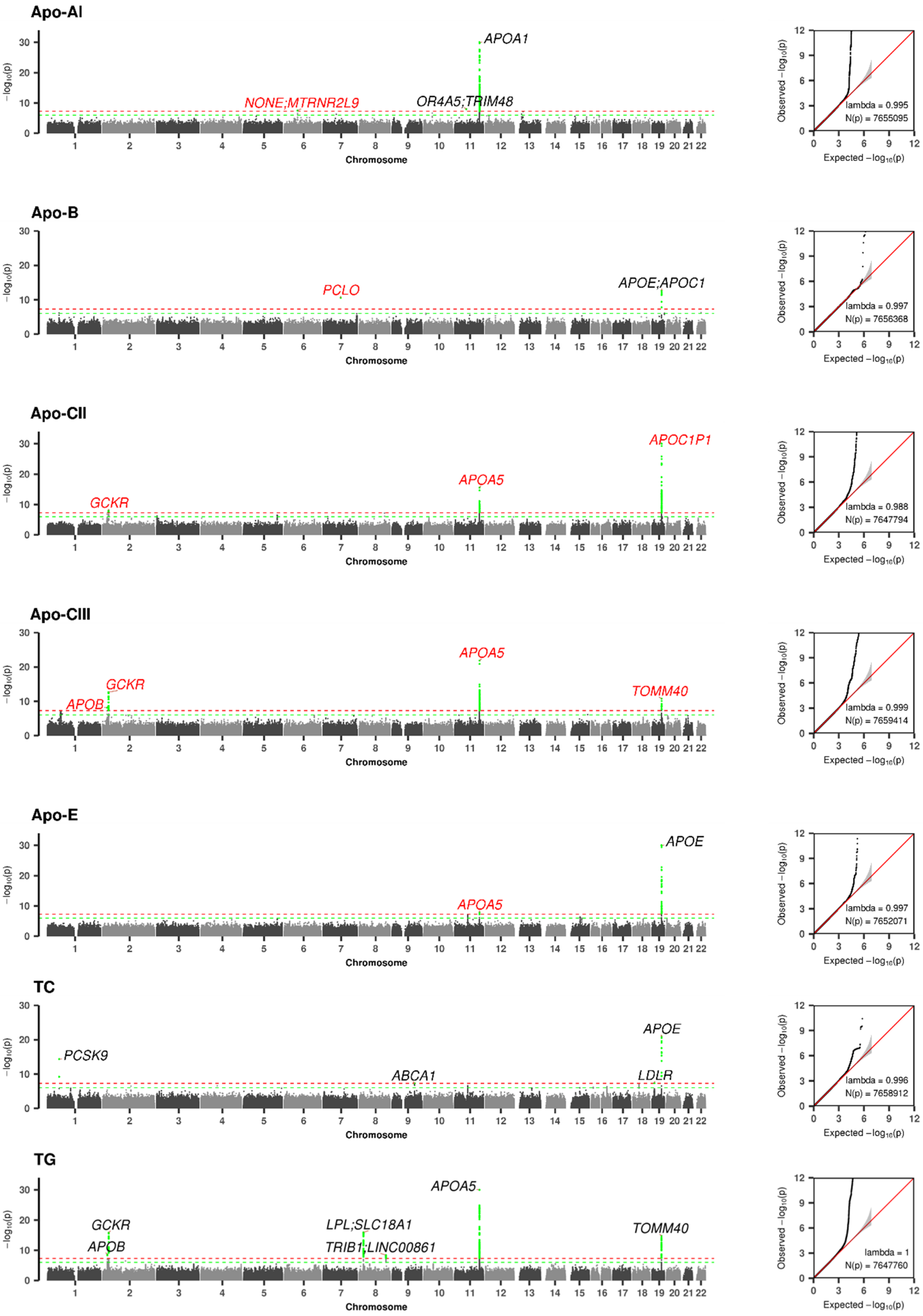

Apo-AI
APOA1
NONE;MTRNR2L9
OR4A5;TRIM48
lambda = 0.995
N(p) = 7655095
Apo-B
PCLO
APOE;APOC1
lambda = 0.997
N(p) = 7656368
Apo-CII
GCKR
APOA5
APOC1P1
lambda = 0.988
N(p) = 7647794
Apo-CIII
APOB
GCKR
APOA5
TOMM40
lambda = 0.999
N(p) = 7659414
Apo-E
APOA5
APOE
lambda = 0.997
N(p) = 7652071
TC
PCSK9
ABCA1
LDLR
APOE
lambda = 0.996
N(p) = 7658912
TG
GCKR
APOB
LPL;SLC18A1
TRIB1;LINC00861
APOA5
TOMM40
lambda = 1
N(p) = 7647760
Chromosome
Expected −log10(p)
Observed −log10(p)
−log10(p)

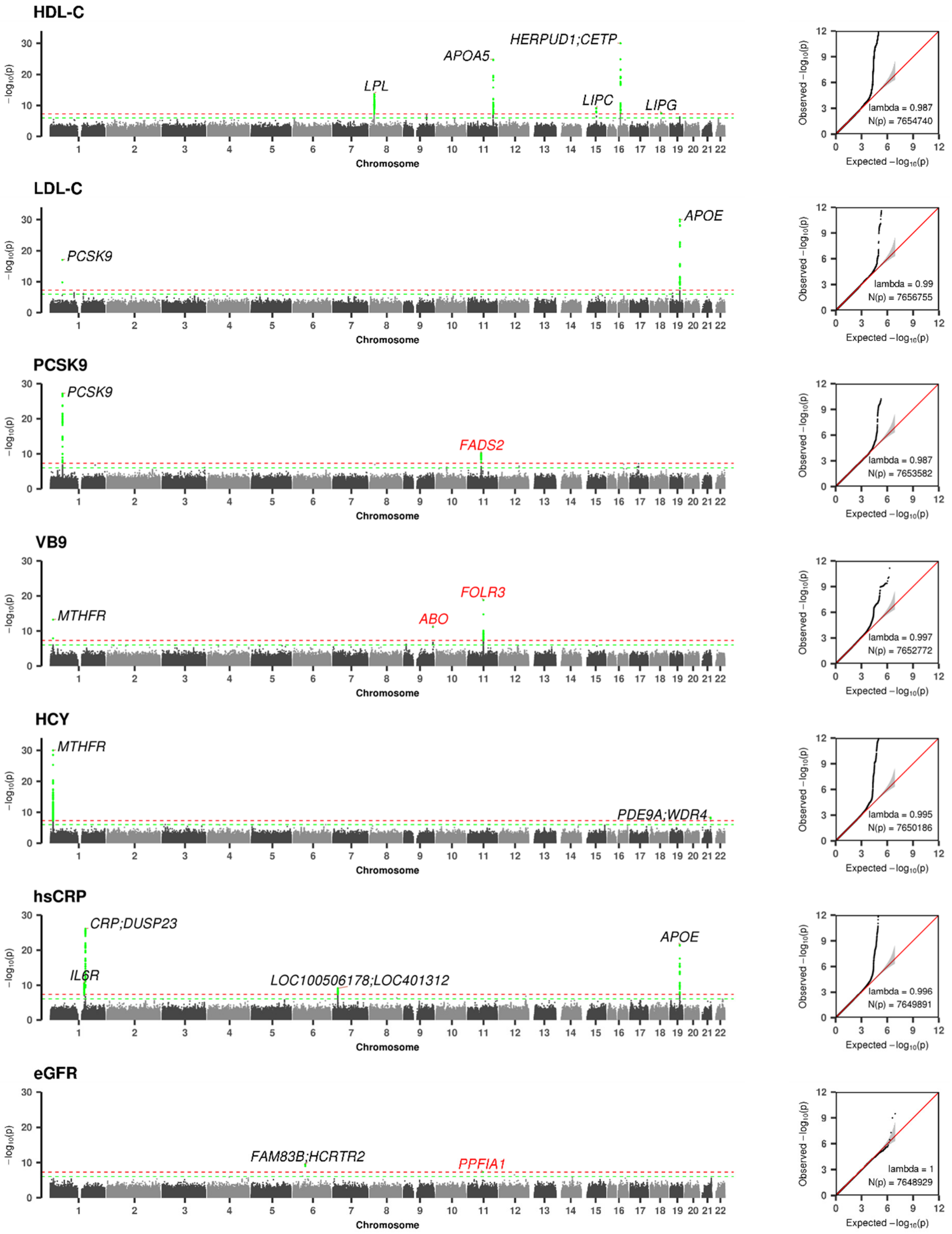

HDL-C
LPL
APOA5
HERPUD1;CETP
LIPC
LIPG
lambda = 0.987
N(p) = 7654740
LDL-C
PCSK9
APOE
lambda = 0.99
N(p) = 7656755
PCSK9
PCSK9
FADS2
lambda = 0.987
N(p) = 7653582
VB9
MTHFR
ABO
FOLR3
lambda = 0.997
N(p) = 7652772
HCY
MTHFR
PDE9A;WDR4
lambda = 0.995
N(p) = 7650186
hsCRP
CRP;DUSP23
IL6R
LOC100506178;LOC401312
APOE
lambda = 0.996
N(p) = 7649891
eGFR
FAM83B;HCRTR2
PPFIA1
lambda = 1
N(p) = 7648929
Chromosome
−log10(p)
Observed −log10(p)
Expected −log10(p)

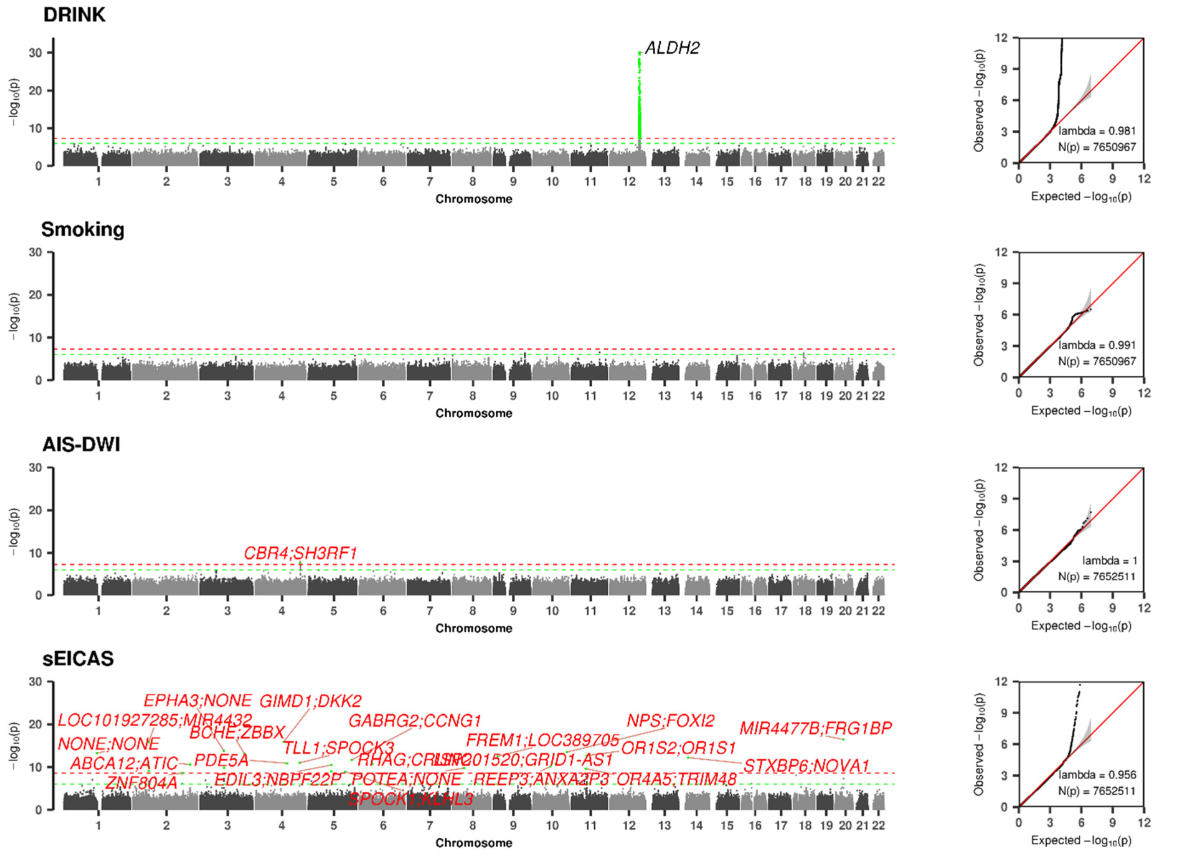

Fig. S19 Manhattan and quantile-quantile (Q-Q) plots for the single variant genome-wide association test for the 18 traits.

In each Manhattan plot, the red dashed line shows *P* value of 5.0×10^{-8} except for the last trait sEICAS, for which the red dashed line shows *P* value of 2.78×10^{-9}. The green dashed line shows *P* value of 10^{-6}.

In Q-Q plots, lambda is the inflation factor of genomic control in GWAS. N (p) shows the number of loci in the GAWS. The area shaded in gray indicates the 95% confidence interval under the null. The red line in each plot represents an idealized case where the theoretical test statistics quantile matches the simulated test statistic quantile.

Abbreviations: AIS-DWI: DWI- positive acute ischaemic stroke. Apo-AI: Apolipoprotein-AI. Apo-B: Apolipoprotein-B. Apo-CⅡ: Apolipoprotein-CⅡ. Apo-CⅢ: Apolipoprotein-CⅢ. Apo-E: Apolipoprotein-E. DRINK: Heavy Drinking. eGFR: baseline estimated glomerular filtration rate. HCY: baseline homocysteine. HDL-C: baseline high-density lipoprotein cholesterol. hsCRP: baseline hypersensitive C-reactive protein. LDL-C: baseline low-density lipoprotein cholesterol. PCSK9: baseline proprotein convertase subtilisin-kexin type 9. sEICAS: Symptomatic extra- and intra-cranial Atherosclerotic Stenosis. TC: baseline total cholesterol. TG: baseline triglyceride. VB9: baseline vitamin B9 (Folate).

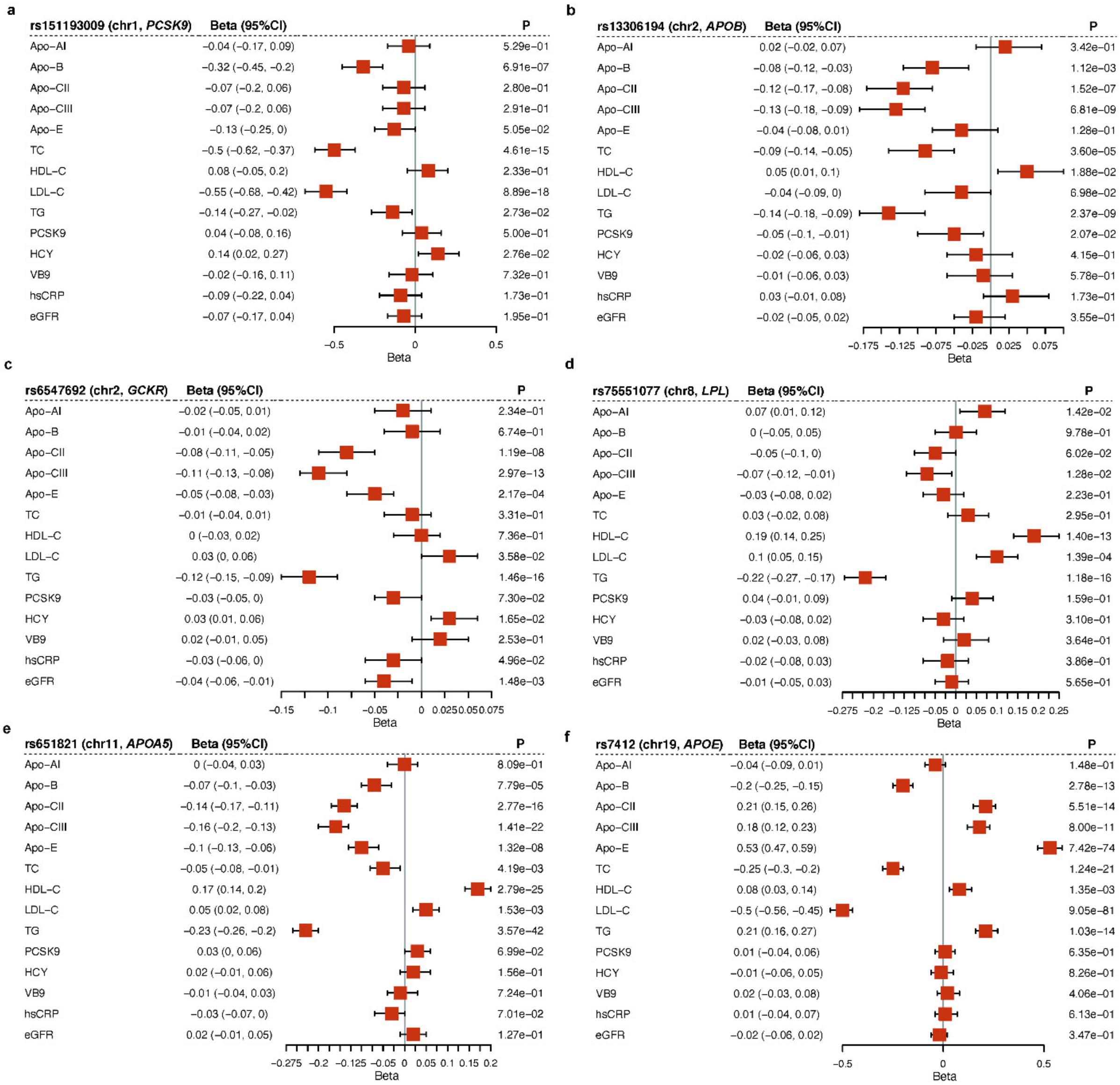

Fig. S20 Forest plot for SNVs with pleiotropic effects on 14 cerebrovascular disease-related biomarkers.

Beta: effect size of association. The 95% confidence interval (CI) was also listed. The chromosome and nearest genes of the associated SNVs were shown.

Apo-AI, Apolipoprotein-AI; Apo-B, Apolipoprotein-B; Apo-CⅡ, Apolipoprotein-CⅡ; Apo-CⅢ, Apolipoprotein-CⅢ; Apo-E, Apolipoprotein-E; TC, baseline total cholesterol; HDL-C, baseline high-density lipoprotein cholesterol; LDL-C, baseline low-density lipoprotein cholesterol; TG, baseline triglyceride; PCSK9, baseline proprotein convertase subtilisin-kexin type 9.

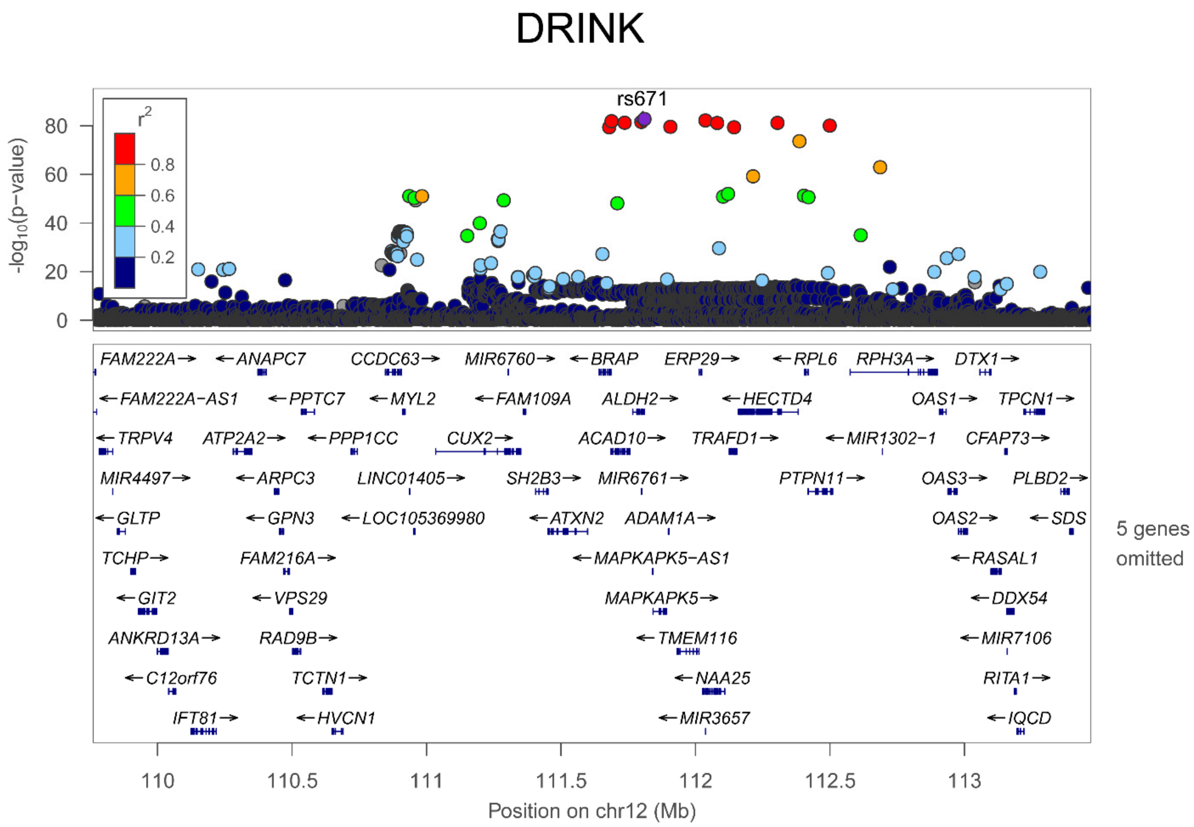

Fig. S21 Locuszoom plot of rs671 associated with heavy drinking behavior. Genomic position is depicted on the x-axis. The left y-axis shows the –log10 of the *P* value. Genetic variants are colored based on their correlation (r^2) with the labeled top SNV, rs671 (purple diamond), which has the smallest *P* value in the region.

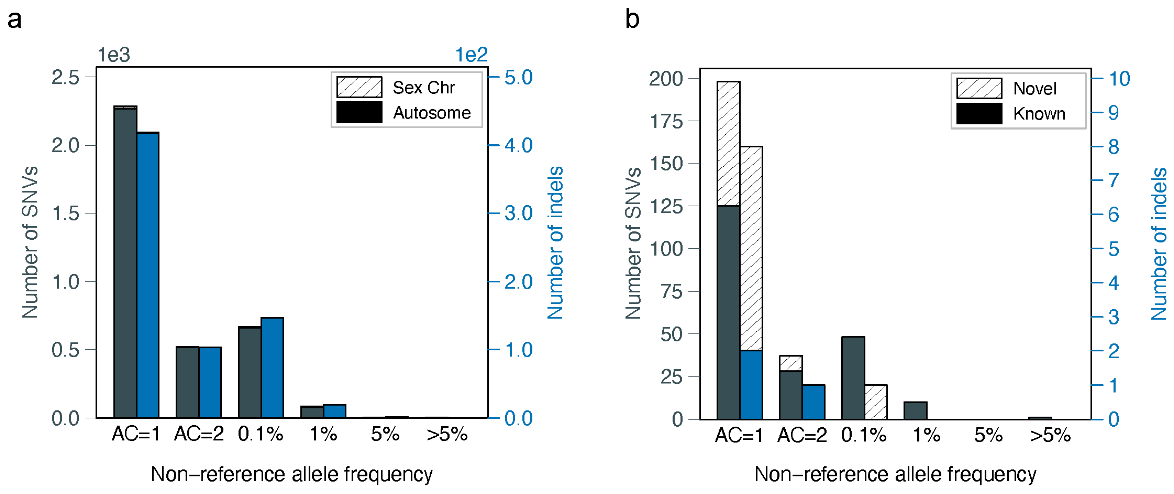

Fig. S22 Summary of ClinVar pathogenic and likely pathogenic variants (P/LP, Materials and methods) in STROMICS and LoF, splicing, and moderate genetic variants in *NOTCH3* gene.

a The number and allele frequency spectrum of ClinVar P/LP variants in STROMICS.

b The number and allele frequency spectrum of LoF, splicing, and moderate genetic variants in *NOTCH3*.

AC, allele count. For detailed definition of LoF, splicing, and moderate genetic variants, see footnotes of Supplementary Table S3. Novel variants are defined by dbSNP (Materials and methods).

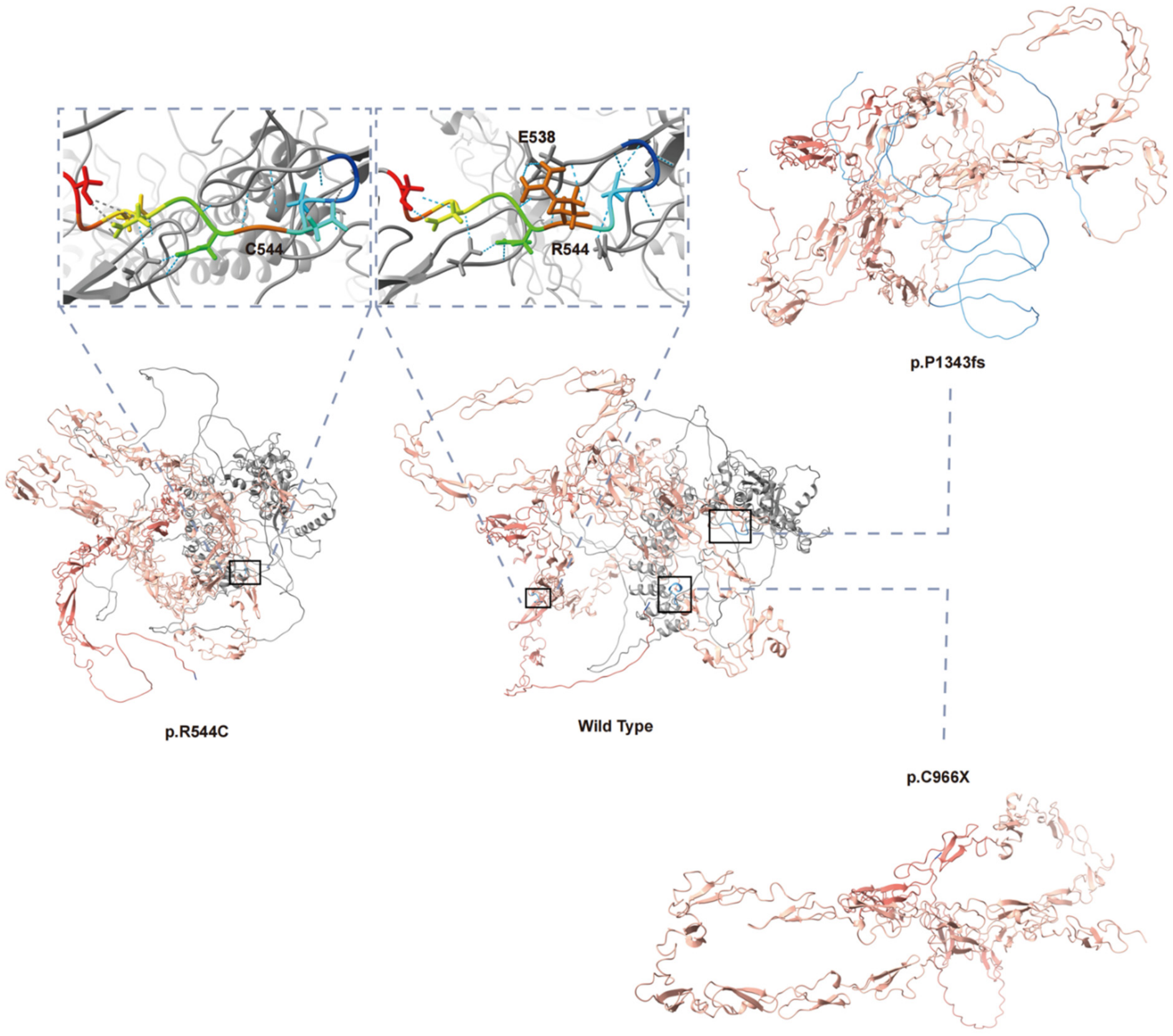

Fig. S23 Protein structure of p.R544C, p.C996X, and p.P1343fs variants in NOTCH3 protein predicted by AlphaFold2. Protein structure of p.R544C, p.C996X, and p.P1343fs variants were matched to the wild-type NOTCH3. The 13th and 14th EGFr domains of the p.R544C variant and the wild-type NOTCH3 were presented with stick form. Cysteine (C) replaced arginine (R) of the 544th amino acid, which interfered with the ionic bond formed between the 544th arginine (R) and 538th glutamic acid (E). The start codon was shown in dark blue. The 1-6 EGFr domains,7-34 EGFr domains, and non-EGFr domains were shown in dark red, light red, and grey, respectively. Additional amino acid sequence after frameshift mutation was shown in sky blue.

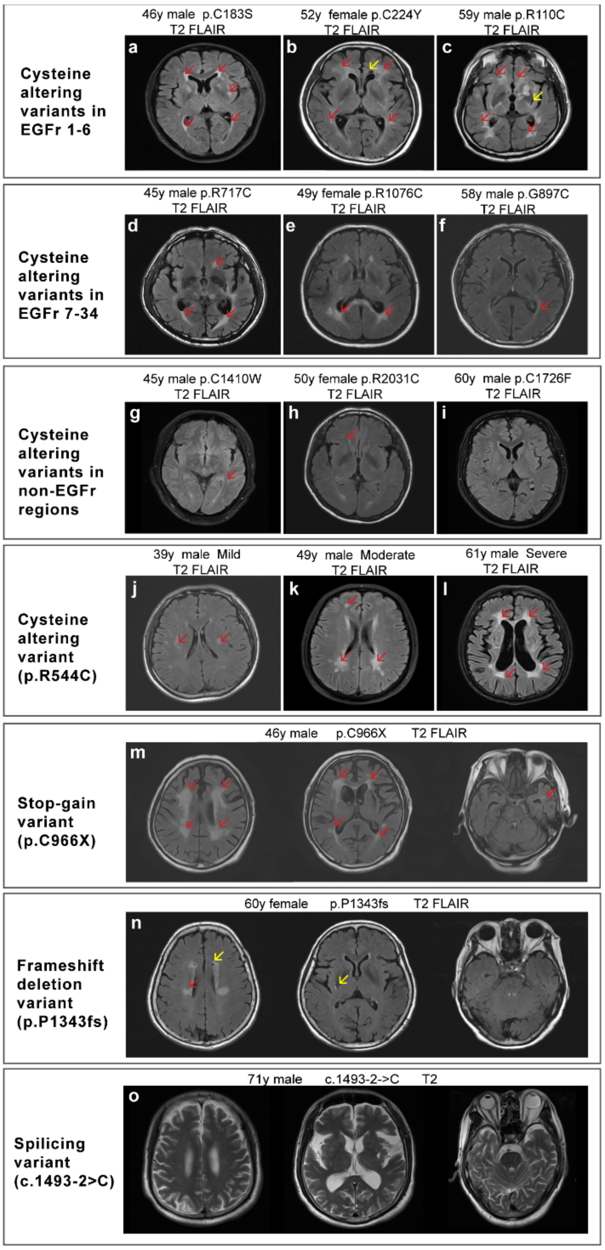

Fig. S24 Neuroimaging of CADASIL-susceptible variant carriers in STROMICS.

a-i Brain MRI T2-fluid-attenuated inversion recovery (T2 FLAIR) images of three representative cases with cysteine (Cys)-altering variant in EGFr domain 1-6, 7-34, and non-EGFr domains, respectively.

j-l Brain MRI T2 FLAIR images of representative patients carrying the p.R544C mutation with mild, moderate, and severe white matter hyperintensity (WMH) load.

m-o Brain MRI T2 FLAIR or T2W images of carriers with the stop-gain variant (p.C966X), frameshift deletion variant (p.P1342fs), and splicing variants (c.1493-2>C). Red arrows indicated WMH. Yellow arrows indicated lacunes. Severity of the WMH load was calculated by the summed score of periventricular hyperintensity (PVH) and deep white matter hyperintensity (DWMH), and was categorized as mild (WMH score < 3), moderate (3-5), or severe (> 5).

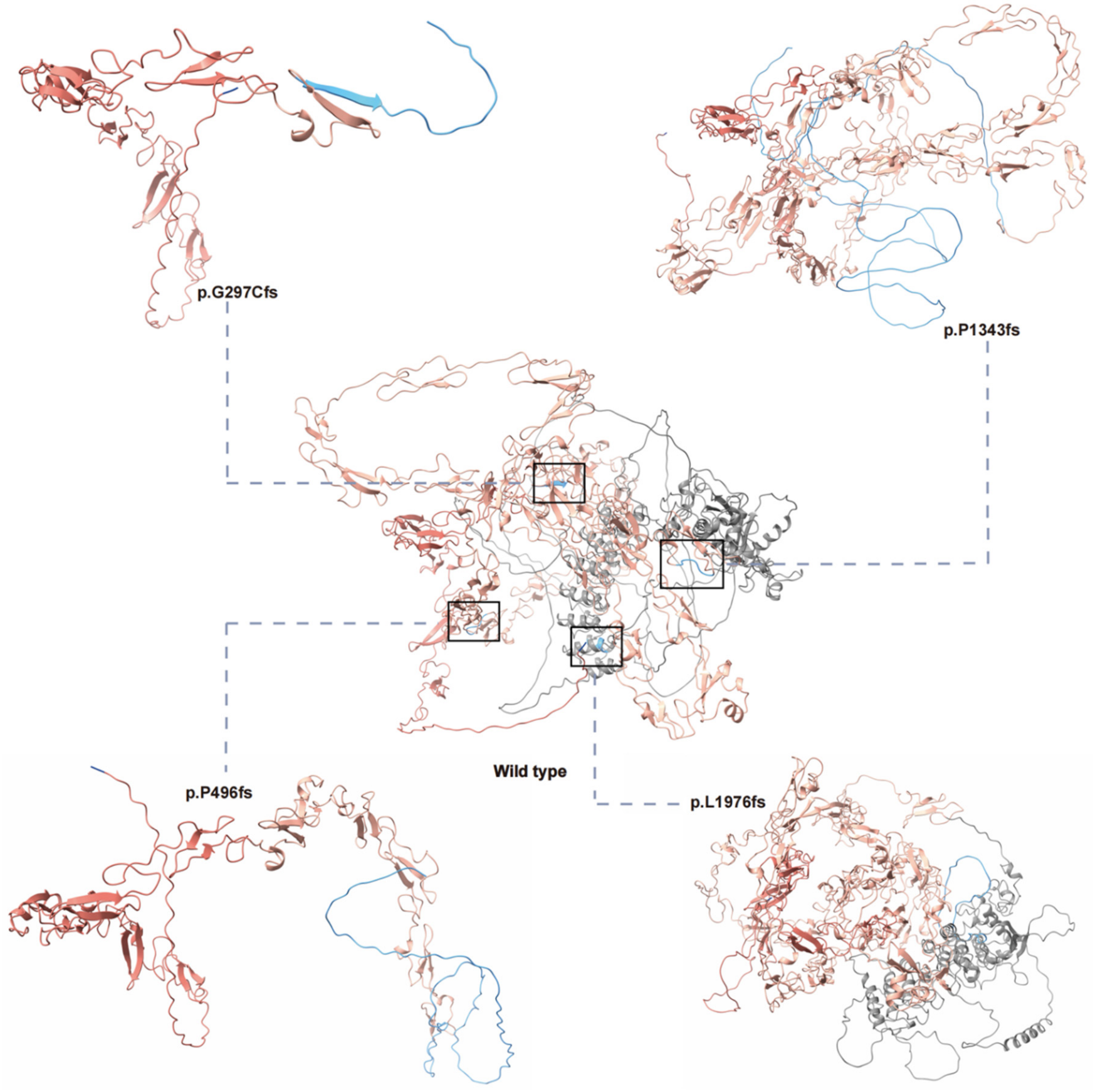

Fig. S25 Protein structure of the four frameshift variants in NOTCH3 protein predicted by AlphaFold2.

Protein structures of the four frameshift variants were matched to the wild-type NOTCH3. The start codon was shown in dark blue. The 1-6 EGFr domains, 7-34 EGFr domains, and non-EGFr domains were shown in dark red, light red, and grey respectively. Additional amino acid sequence after frameshift mutation was shown in sky blue. The black boxes marked the position of the mutant site of the four frameshift variants at the wild-type protein.
